# Receptor-Mediated Shuttling of a D‑Amino Acid Peptide Achieves High Nanomolar Cytosolic Concentrations

**DOI:** 10.1021/jacs.5c12876

**Published:** 2025-12-21

**Authors:** Moritz List, Annette G. Beck-Sickinger

**Affiliations:** Institute of Biochemistry, Faculty of Life Sciences, 9180 Leipzig University, Leipzig 04103, Germany

## Abstract

Delivery of therapeutic peptides and proteins to the cytosol is of great interest due to their ability to inhibit intracellular protein–protein interactions, which are mostly deemed undruggable by small molecules. Internalization into the endosomal pathway is possible by receptor-targeted approaches; however, endosomal escape is inefficient, and its quantification is challenging. To improve our current understanding of cytosolic delivery, we performed comprehensive studies on a receptor-mediated shuttle system based on the chemokine-like receptor 1 (CMKLR1). As a model cargo, PMIγ was used, a known D-amino acid peptide antagonist of the MDM2/p53 interaction, which survives the harsh conditions in the endocytic pathway. Fluorescence correlation spectroscopy (FCS) was used to demonstrate that biologically meaningful cytosolic concentrations (>100 nM) can be reached by receptor-mediated shuttling, even when no endosomal escape enhancing strategies are used. Attachment of the pH-responsive endosomal escape peptide (EEP) hsLMWP further improved cytosolic delivery but also induced cellular toxicity at higher concentrations. Additionally, the EEP activity was likely limited by its fast degradation after internalization. Intracellular biological activity was confirmed using bioluminescence resonance energy transfer (BRET) studies, which demonstrate binding to MDM2 and inhibition of the p53/MDM2 interaction. This study highlights the potential of receptor-mediated shuttling for cytosolic delivery of therapeutic peptides and provides new insights into achievable intracellular concentrations, advancing the field of peptide therapeutics and drug delivery.

## Graphical abstract

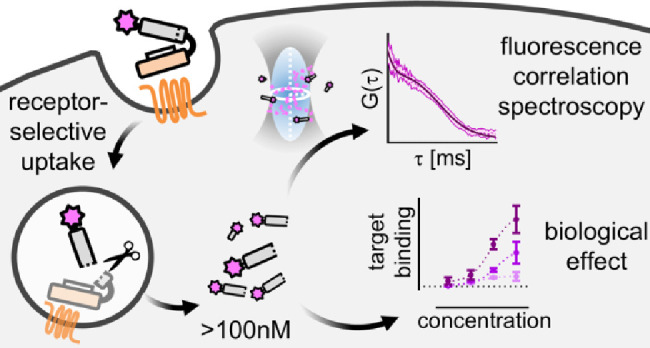

## Introduction

Therapeutic peptides addressing intracellular targets are of great interest due to their ability to interfere with protein–protein interactions.^1^ While receptor-mediated shuttling promises a valuable tool for the delivery of peptides into target cells, reaching the cytosol has proven particularly elusive, as endocytosis generally leads to endosomal entrapment and inefficient endosomal escape.^2^ Quantifying cytosolic delivery of peptide cargos after receptor-mediated uptake is also fundamentally difficult and has been a major roadblock for the advancement of this strategy. Inefficient endosomal escape makes distinguishing between large amounts of peptides trapped within endosomal vesicles and those freely diffusing in the cytosol a major experimental hurdle. Various cell-based assays have been reported in recent years^3–9^ ; however, most of them provide indirect or amplified read-outs and often have other issues that makes them unsuitable when investigating receptor-mediated uptake. Especially proteolytic degradation of detection tags is critical when peptides are taken up into the endo/lysosomal pathway. As such, the recently proposed split luciferase endosomal escape quantification assay^10^ or the biotin ligase assay^9^ use tags consisting of L-amino acids, whose degradation in the lysosomes interferes with their readout. Fluorescence correlation spectroscopy (FCS) offers a different option to directly quantify the concentration of fluorescently labeled peptides in the cytosol of living cells.^11–14^ Here, fluctuations in fluorescence are detected, as molecules diffuse through the focal volume of a confocal microscope placed in the cytosol. Autocorrelation of the fluorescence intensity over time can then be used to extract concentration and diffusion dynamics of the measured molecules.^15^ While this method has very low throughput (∼1 min per measurement in a single cell), it is the only method that directly measures an absolute value of the cytosolic concentration. Until now, FCS has only been used to quantify delivery of membrane permeable compounds or cargo shuttled by cell-penetrating peptides (CPPs). We have now developed a strategy to quantify cytosolic delivery of peptide cargos by a targeted shuttle system for the first time (Scheme 1).

**Scheme 1:**
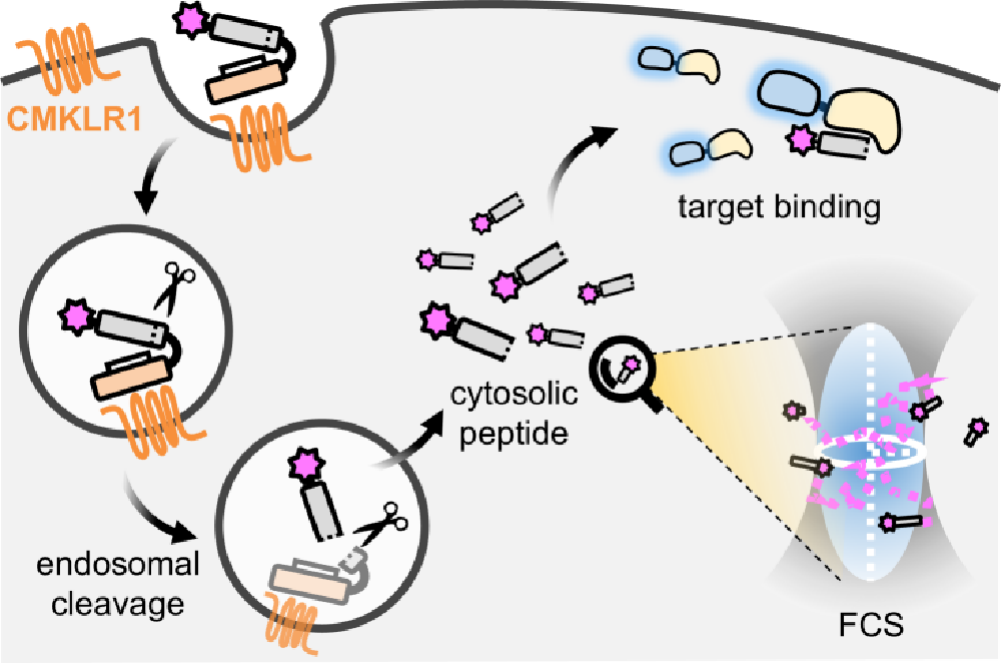
Proposed Uptake Mechanism of D-Peptides by the Chemokine-like Receptor 1 (CMKLR1)^a^

a Cytosolic concentrations were determined by fluorescence correlation spectroscopy (FCS) and confirmed by bioluminescence energy transfer (BRET) studies after binding to the intracellular target protein.

When sufficiently stabilized against proteolysis, peptides are promising cargos for such a shuttle. As peptides can be chemically synthesized, stabilization can be achieved by cyclization^16^ or the use of unnatural amino acids.^17^ Recently, intracellular activity has been demonstrated when the membrane impermeable proapoptotic D-peptide ^D^KLA was conjugated to iRGD, a ligand for different integrins and the neuropilin-1 receptor, both of which are overexpressed on many cancers.^18^

To improve the rate of endosomal release, various endosomal escape peptides (EEPs) have been reported recently.^19,20^ Histidine-switching low molecular weight protamine (hsLMW) enabled efficient endosomal release after receptor-mediated endocytosis when conjugated to the HER2-targeting antibody trastuzumab.^11,21^ However, until now, actual intracellular concentrations that can be reached by these novel EEPs have not been reported. In this work, FCS is used to quantify the delivery of a D-amino acid peptide by a receptor-based shuttle system. Proteolytically stable peptides have been shown to reside longer in the cytosol and thereby reach higher concentrations, which facilitates successful detection.^22,23^ Thus, the D-peptide PMIγ, a known antagonist of MDM2 that binds with a *K*_d_ of 52.8 nM, was chosen as a model cargo.^24^ MDM2 is an E3 ligase that regulates p53 levels through ubiquitination and plays a key role in the inactivation of the apoptotic p53 pathway in many cancer types.^25–27^ By restoring p53 activity, inhibitors of MDM2 and its homologue MDMX are potentially valuable therapeutics for cancer. PMIγ, however, is not membrane permeable, and conjugation to CPPs was generally found to be ubiquitously cytotoxic, independent of p53 status. The need for a better delivery method and its resistance to lysosomal proteases made PMIγ an ideal cargo for a receptor-mediated delivery system. For shuttling, the chemokine-like receptor 1 (CMKLR1) was targeted. This G protein-coupled receptor is expressed in the immune system^28,29^ and overexpressed in various cancer types, including lung,^30^ prostate,^31^ and colorectal cancer.^32^ Recent studies highlight the potential of CMKLR1 as a therapeutic target, with the development of a positron emission tomography tracer^33^ and a peptide-drug conjugate^34^ based on analogues of the efficiently internalizing nona-peptide ligand chemerin-9.^35^ Here, a proteolytically more stable cyclic variant with a disulfide bridge and a C-terminal azide termed cyclic chemerin-9 (N_3_-cC9, peptide **1**) was used and conjugated to PMIγ using copper-catalyzed azide–alkyne cycloaddition (CuAAC).^36^ This provided a modular peptide platform, in which either the cargo or the targeting ligand could be easily exchanged. Between PMIγ and cC9, a valine-citrulline linker was included to allow a release in the endosome by the protease cathepsin B to yield peptide **2** (PMIγ-cC9).^37^ Peptide **3** with enhanced endosomal release was generated by the N-terminal attachment of the EEP hsLMWP (hsLMWP-PMIγ-cC9). Finally, the cationic CPP nona-D-arginine was conjugated to PMIγ to obtain the nonselectively internalizing peptide **4** (r_9_-PMIγ). Each peptide was prepared in a fluorescently labeled variant with the far-red fluorophore sulfo-Cy5 to give peptides **2Cy**, **3Cy**, and **4Cy**. To prevent proteolytic release, positions for attachment of the fluorophore were chosen carefully so that only D-amino acids and unnatural building blocks connect Cy5 and PMIγ.

## Results and Discussion

### Peptide Characterization

Peptides (Table 1) were synthesized by solid phase peptide synthesis using the Fmoc/tBu strategy. Peptides **2**, **2Cy**, **3**, and **3Cy** were first prepared with a C-terminal alkyne and conjugated to azide-labeled cyclic chemerin-9 (peptide **1**) by CuAAC (Figure S1). After purification, peptides were characterized by ESI-Orbitrap MS, and homogeneity was demonstrated by RP-HPLC on two different columns (Figure S2). Schematic structures of the fluorescently labeled peptides **2Cy**, **3Cy**, and **4Cy** are shown in Figure 1A. All three constructs show binding to MDM2 in a fluorescence polarization assay with a *K*_d_ of 90 nM for both **2Cy** and **3Cy** and 57 nM for **4Cy**. Overall, the novel conjugates are only slightly less potent compared to a *K*_d_ of 52.8 nM that was reported for unmodified PMIγ.^24^

**Table 1:** Sequence and Analytical Data of the Peptides Discussed in This Work^a^

	name	sequence	*M*_mono_ [Da]	*M*_obs_ [*m*/*z*]	purity [%]
**1**	N_3_-cC9	Ac–K(N_3_)YFP-**hcys**-QFAF**C**–OH	1332.54	1333.55	>95%
**2**	PMIγ-cC9	dwwplafeallrGEV-Cit-(EG)_3_-Pra(cC9)-NH_2_	3644.74	3645.76	>95%
**2Cy**	Cy5-PMIγ-cC9	Cy5-(EG)_3_-dwwplafeallrGEV-Cit-(EG)_3_-Pra(cC9)-NH_2_	4472.05	4473.08	>95%
**3**	hsLMWP-PMIγ-cC9	GSVSHHHHHHGGHHHH-(EG)_3_-dwwplafeallrGEV-Cit-(EG)_3_-Pra(cC9)-NH_2_	5605.62	5606.65	>95%
**3Cy**	Cy5-hsLMWP-PMIγ-cC9	GSVSHHHHHHGGHHHH-(EG)_3_-k(Cy5-(EG)_3_)dwwplafeallrGEV-Cit-(EG)_3_-Pra(cC9)-NH_2_	6561.02	6562.05	>95%
**4**	r_9_-PMIγ	NH_2_-rrrrrrrrr-(EG)_3_-dwwplafeallrGEV-Cit-(EG)_3_-Pra-NH_2_	3863.20	3864.23	>95%
**4Cy**	Cy5-r_9_-PMIγ	Cy5-(EG)_3_-rrrrrrrrr-(EG)_3_-dwwplafeallrGEV-Cit-(EG)_3_-Pra-NH_2_	4690.51	4691.54	>90%

a Small letters represent the corresponding D-amino acid. Purity was determined on two different RP-HPLC columns. MS and HPLC analysis are displayed in Figure S2. Cysteines printed in bold are cyclized by a disulfide bridge. cC9 = cyclic chemerin-9, Cy5 = sulfo-cyanine5, K­(N_3_) = azidolysine, hcys = d-homocysteine, Cit = citrulline, (EG)_3_ = triethylenglycol, Pra = propargylglycine.

**Figure 1:**
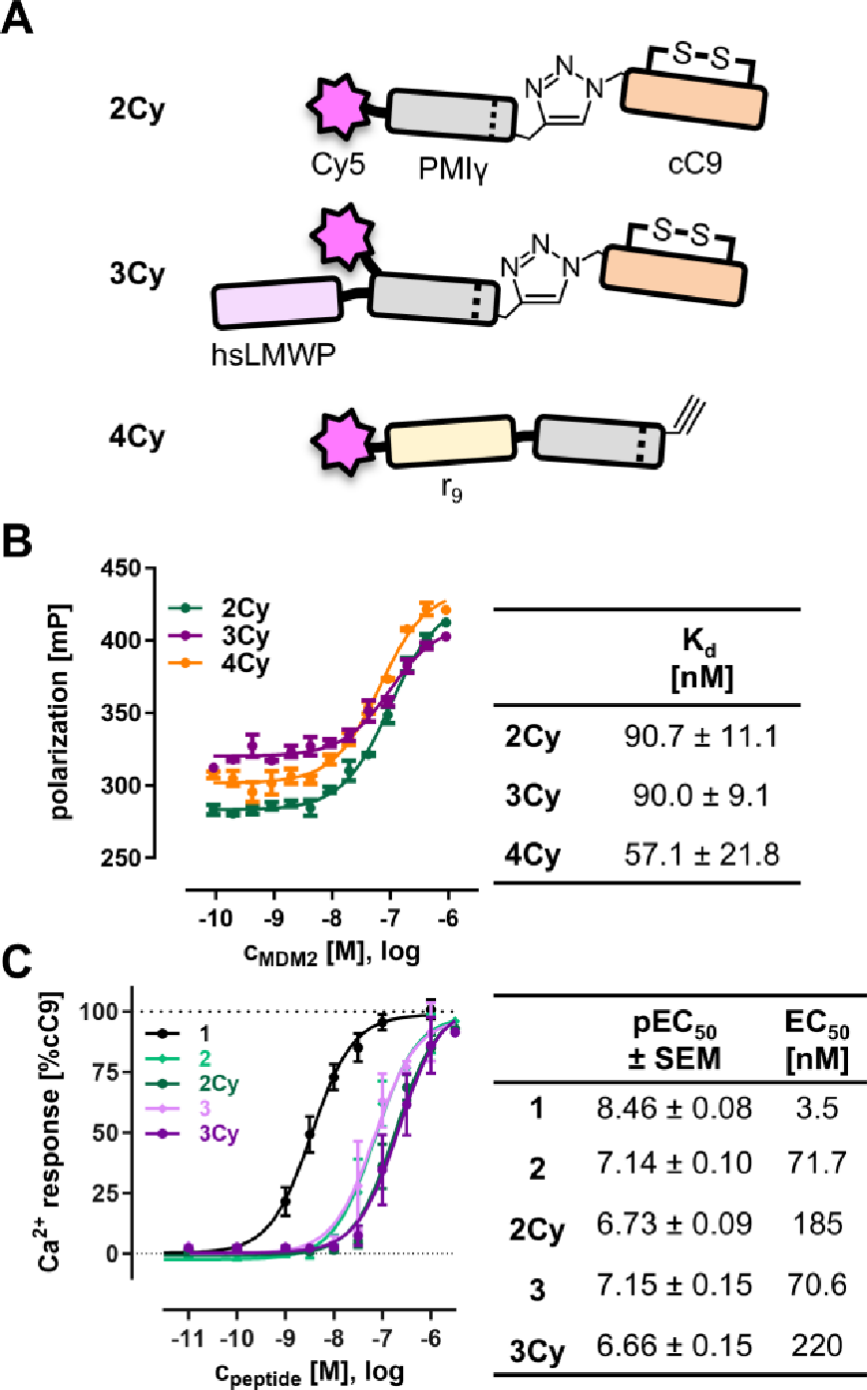
Structure and activity of peptides. (A) Schematic structure of peptides discussed in this work. (B) Fluorescence polarization measurement of 20 nM Cy5-labeled peptides binding to recombinant human GST-MDM2. (C) Activity of the cyclic chemerin-9 (cC9) derivatives at CMKLR1 tested in a Ca^2+^ flux assay. Data points represent the mean ± SEM of at least two independent experiments performed in duplicate.

For cell-based assays, the immortalized noncancer cell line HEK293 was chosen. HEK293 cells endogenously express both p53 ^38^ and MDM2 ^39^; however, they do not undergo cell death due to MDM2 inhibition as can be shown from the noncytotoxic response to the small molecule MDM2 antagonist nutlin-3a (Figure S3). Thus, cytosolic uptake of PMIγ is not expected to result in apoptosis due to MDM2 inhibition either, which makes the cells well-suited for uptake studies. Accordingly, the peptides **2** and **2Cy** showed no signs of cytotoxicity, even after 72 h of incubation on HEK293_CMKLR1-eYFP cells (Figure S4). The hsLMWP-containing peptides **3** and **3Cy** were tolerated slightly worse at concentrations above 1 μM, potentially due to unspecific membrane disruption due to the histidine-rich hsLMWP sequence.^21^ The CPP-containing peptides **4** and **4Cy** were even more cytotoxic at high concentrations, which has been reported for other CPP-PMIγ conjugates before.^24^ Thus, the CMKLR1-targeting peptides **2**, **2Cy**, **3**, and **3Cy** were focused on uptake studies.

Initially, a Ca^2+^ flux assay was used to investigate the activity at CMKLR1.^35^ Here, the unlabeled peptides **2** and **3** both show similar nanomolar activity with EC_50_ of 71.7 and 70.6 nM (Figure 1C), irrespective of their apparent differences in molecular size. Interestingly, introduction of the fluorophore led to a further ∼ 3-fold loss of activity with EC_50_ of 185 and 220 nM for **2Cy** and **3Cy**, respectively. Electrostatic repulsion between sulfo-cyanine5 and a negatively charged patch on the receptor surface could explain the lower potency of the labeled peptides.^40^

### Peptide Internalization and Cytosolic Detection

Both fluorescently labeled peptides internalized efficiently into HEK293 cells transfected with CMKLR1 and punctate Cy5-fluorescence inside the cells was observed, hinting at endosomal entrapment of the peptides (Figure 2). While **2Cy** did not internalize into cells without CMKLR1, **3Cy** showed very faint intracellular signals. Like the observed cytotoxicity, unspecific internalization could have been driven by membrane association through the histidine-rich hsLMWP sequence.

**Figure 2:**
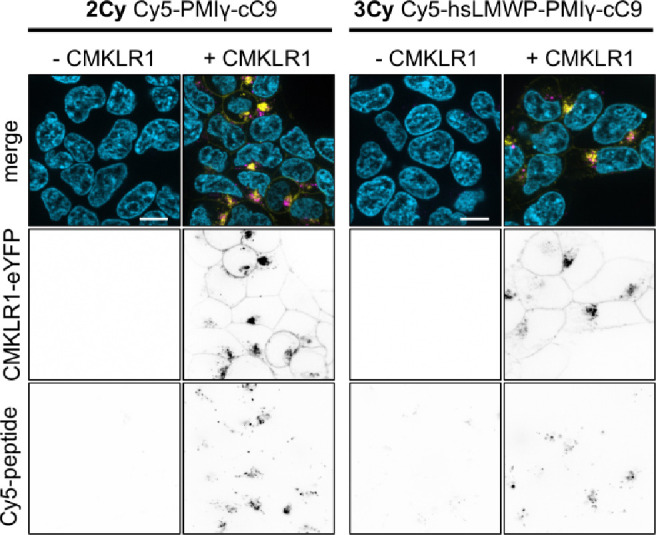
Internalization of cyclic chemerin-9 conjugates. Live cell fluorescence microscopy of HEK293 and HEK293 cells stably expressed CMKLR1-eYFP. Cells were incubated with 1 μM Cy5-labeled peptide for 6 h before imaging. Blue = Hoechst33342, yellow = CMKLR1-eYFP, magenta = Cy5-peptide, scale bar ≙ 10 μm.

Peptide uptake was quantified in the lysate of stably transfected HEK293 cells as well as HMEC-1, MDA-MB-231, and THP-1 cells that endogenously express CMKLR1 (Figure S5A).^28,41,42^ Consistent with the observed unspecific membrane activity, **3Cy** showed uptake slightly higher than that of **2Cy** in all cells (Figure S5B). Notably, the observed peptide uptake correlated well with the CMKLR1 expression of all cell lines except HMEC-1, which demonstrated a significantly higher uptake relative to the receptor amount (Figure S5C).

After internalization, peptides enter the endo/lysosomal pathway, where their degradation was investigated by analyzing Cy5-labeled cleavage products in the lysate of HEK293_CMKLR1-eYFP cells (Figure 3A). Here, aggressive degradation was observed for both **2Cy** and **3Cy** and almost no intact peptide was detected in the lysates. Instead, after 15 min and 1 h, respectively, a singular peak, which continuously accumulated over time, was observed for each peptide. Identification by MALDI-ToF-MS revealed that the valine-citrulline linker was quickly cleaved in both peptides, while the PMIγ sequence remained intact. Additionally, in **3Cy**, the C-terminal hsLMWP sequence was completely removed, resulting in a Cy5-labeled fragment of 2400.12 Da for **2Cy** and 2731.33 Da for **3Cy**, respectively. We hypothesize that the effect of hsLMWP on cytosolic delivery might be limited by its low endosomal stability. FCS measurements were performed to directly quantify the amount of fluorescently labeled peptide that reached the cytosol. Briefly, cells were first incubated with peptide **2Cy** or **3Cy**. After extensive washing, cells were placed on a confocal microscope, cytosolic locations distant from regions with strong punctate fluorescence were chosen for the measurements and ten consecutive 5 s fluorescence traces were taken. Invalid measurements were discarded, and the remaining autocorrelation curves averaged (details in Scheme S5). Representative autocorrelation traces measured in the cytosol are shown in Figure 3B. Autocorrelation curves were fitted to obtain concentration, diffusion coefficient *D*, and anomalous coefficient α. Generally, FCS measurements likely overestimate cytosolic concentrations slightly for two reasons: First, the detection limit of FCS in cells is about 1 nM, due to the high amount of background fluorescence from biological structures and cell culture media.^43^ Thus, if cytosolic concentrations were much lower, no valid autocorrelation curves could be fitted and the data point would be excluded from the data set. Second, FCS overestimates concentrations at low signal-to-noise ratios,^44^ as is the case during measurements of low cytosolic concentration in living cells. Therefore, a comparatively long incubation time of 72 h was chosen to allow for high cytosolic accumulation of the peptides.

**Figure 3:**
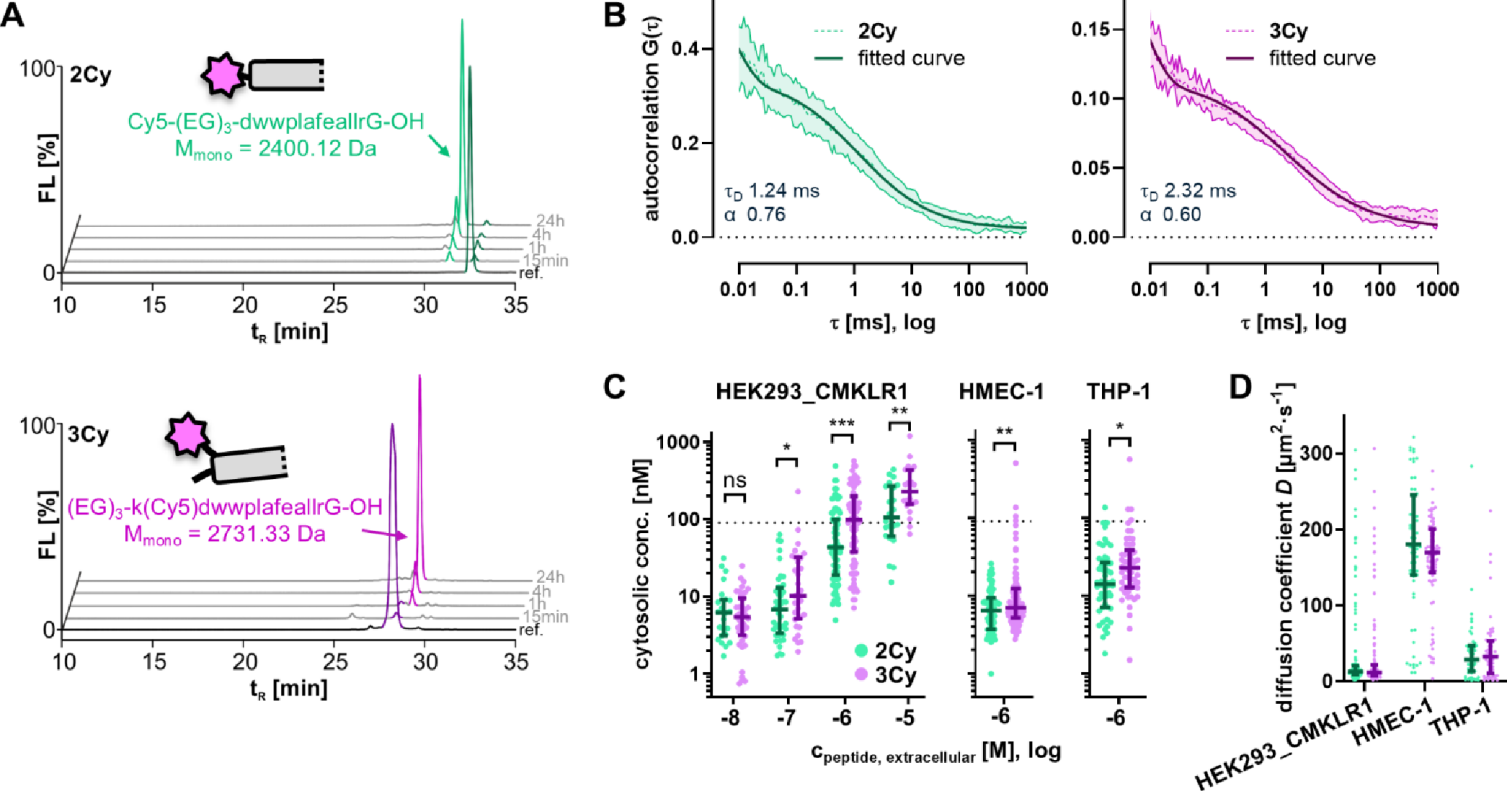
Quantification of Cy5-labeled peptides in the cytosol. (A) RP-HPLC (λ_ex_ = 640 nm, λ_em_ = 670 nm) of cleavage products of Cy5-labeled peptides in the lysate of HEK293_CMKLR1-eYFP cells after incubation with 1 μM peptide for different durations. Main cleavage products were identified by MALDI-ToF-MS and their structures are shown. (B) Representative fluorescence correlation spectroscopy (FCS) autocorrelation curves of Cy5-labeled peptides measured in the cytosol of HEK293_CMKLR1-eYFP cells after 72 h of incubation. Measured curves were fitted with a model for hindered 3D diffusion and diffusion time *t*_D_ and anomalous coefficient α were calculated from the fit. Autocorrelation data represent mean ± SD of up to 10 consecutive 5 s measurements in a single cell. (C) Intracellular concentrations of Cy5-labeled peptides after 72 h incubation calculated from the fitted autocorrelation curves obtained in HEK293_CMKLR1-eYFP, HMEC-1, and THP-1 cells. Each data point corresponds to FCS measurements from the cytosol of a single cell. Median with the interquartile range is shown. Data from three independent experiments. Statistical comparison by log-normal unpaired *t* test. ***p ≤ 0.001, **p ≤ 0.01, *p ≤ 0.05, ns – not significant. The dashed line corresponds to the measured *K*_d_ value (90 nM) of the PMIγ/MDM2 interaction. (D) Calculated diffusion coefficients from the fitted autocorrelation curves. Median with the interquartile range is shown.

The measured cytosolic concentrations were distributed roughly log-normally and clearly correlated with the extracellularly applied concentration for stably transfected HEK293_CMKLR1-eYFP cells (Figure 3C, descriptive statistics in Table S7). Notably, the cytosolic concentrations in many cells surpassed the measured *K*_d_ of the PMIγ/MDM2 interaction (90 nM). At 1 μM extracellular peptide concentration, **2Cy** reached a median cytosolic concentration of 44.6 nM (interquartile range (IQR) 19.0–99.4 nM). **3Cy** achieved a significantly higher median concentration of 98.8 nM (IQR 37.8–199 nM), hinting at an increase in endosomal escape efficiency due to hsLMWP. The same trend was seen with 10 μM of the peptides, where median cytosolic concentrations of 106 nM (IQR 60.2–269 nM) and 228 nM (IQR 157–435 nM) were measured for **2Cy** and **3Cy** respectively. However, consistent with the observed cytotoxicity (Figure S4), cells incubated with 10 μM **3Cy** showed signs of cellular stress (data not shown), and FCS data quality was overall lower, only giving viable measurements in about 25% of cells, compared to the usual 50% for the other conditions. Cytosolic delivery of **2Cy** and **3Cy** was also detected by FCS in endogenously CMKLR1-expressing HMEC-1 and THP-1 cells (Figure 3C). In contrast, no successful measurements were obtained in MDA-MB-231 cells, likely because of their lower level of CMKLR1 expression (Figure S5A). Overall, cytosolic concentrations were lower than those in the stably transfected cells, and an increased delivery of **3Cy** was observed again. It should be noted that the uptake data represents an end point measurement after 72 h incubation and does not account for a variety of dynamic processes such as cell growth, autophagy, or exocytosis, which likely influence both peptide accumulation and loss over this longer timespan.^45^

### Hindered Cytosolic Diffusion

In addition to the cytosolic concentration, FCS measurements provide diffusion coefficients of the measured species, which reveal differences between the different cell lines (Figure 3D). In HEK293_CMKLR1-eYFP cells, the median intracellular diffusion coefficients were surprisingly low at 13.1 μm^2^/s (IQR 9.3–20.7 μm^2^/s) for **2Cy** and 11.3 μm^2^/s (IQR 7.3–21.7 μm^2^/s) for **3Cy** – about 14-fold lower compared to the intact peptides in solution (Table S8). Diffusion coefficients in THP-1 cells were higher at 28.9 μm^2^/s (IQR 13.2–47.0 μm^2^/s) and 32.6 μm^2^/s (IQR 10.2–53.2 μm^2^/s), respectively, representing a 5-fold decrease compared to an extracellular measurement. Surprisingly, measurements obtained in the cytosol of HMEC-1 cells gave diffusion coefficients of 180 μm^2^/s (IQR 140–245 μm^2^/s) and 169 μm^2^/s (IQR 143–201 μm^2^/s), respectively, which was higher than those obtained from the intact peptides in solution (Table S8). Considering diffusion coefficients correlate inversely with the cube root of molecular weight, these results align with the predicted diffusion coefficients of the ∼2.5 kDa intracellular cleavage products of **2Cy** and **3Cy** rather than the intact peptides.^46,47^ Compared to diffusion in dilute solutions, diffusion in the cytosol is assumed to be slowed due to hindrance by the crowded molecular environment. For reference, intracellular FCS measurements of multiple rhodamine-labeled CPPs and stapled peptides (1.9–5.0 kDa) in HeLa cells have been reported with diffusion coefficients between 43.2 and 82.9 μm^2^/s, about 3 to 5-fold lower compared to extracellular measurements.^48^ In similar intracellular measurements of rhodamine-labeled SnapTag-CPP conjugates (20–24 kDa) in Saos-2 cells, a 6 to 14-fold reduction in diffusion coefficients has been reported.^11^ Thus, the high diffusion coefficients in HMEC-1 cells seem to represent almost unhindered diffusion. These results might also explain the more efficient uptake in these cells (Figure S5C), which could be the result of faster internalization, sorting and recycling processes. Contrarily, the exceptionally low diffusion coefficients observed in HEK293_CMKLR-eYFP and THP-1 cells could hint at intracellular binding of the peptides to MDM2 (55.2 kDa), which would significantly increase the hydrodynamic radius and thereby slow down diffusion.

### Intracellular Binding of PMIγ to MDM2

To investigate binding of the cytosolic peptides to intracellular MDM2, a bioluminescence energy transfer (BRET) assay using NanoLuc-tagged MDM2 and Cy5-labeled peptides **2Cy** and **3Cy** was performed. In agreement with the FCS measurements, significantly elevated BRET was observed in CMKLR1-expressing cells after 72 h of incubation with 1 or 10 μM of either peptide (Figure 4A). Without CMKLR1, only a minor signal was seen for **2Cy**, while **3Cy** gave a strong BRET at 10 μM, again likely correlated with its unspecific membrane activity. Measurements after shorter incubation times of 24 and 48 h gave similar results, and accumulation of the peptides over time was clearly seen here (Figure S9). Interestingly, in cells without CMKLR1, no such accumulation was observed. Rather, the unspecific BRET signal remained constant over time and is more pronounced with **3Cy**, hinting that it could have arisen from cells with compromised membrane integrity during the measurements. To isolate the receptor-specific effect, ΔBRET values were calculated by subtracting the netBRET from cells without CMKLR1 from cells with CMKLR1 (Figure 4B). Plotting ΔBRET values from measurements at 24, 48, and 72 h highlights the receptor-mediated accumulation of the peptides as well. Even in the ΔBRET representation **3Cy** showed slightly higher signal compared to **2Cy**, suggesting an improved cytosolic delivery due to hsLMWP. To further confirm CMKLR1-selectivity of the accumulation, the BRET experiments were repeated while receptor-mediated uptake was competed by coincubation with 30 μM of the unlabeled peptide **1** (Figure 4C). Now, almost no receptor-specific BRET was detected and over different time points no accumulation was observed (Figure S10). Calculated ΔBRET curves were completely flat, except for 10 μM **3Cy**, thus confirming that the previously observed uptake is indeed CMKLR1-specific (Figure 4D). To confirm that the BRET signal results specifically from PMIγ binding to MDM2, an additional kinetic measurement was performed. Here, 10 μM of the MDM2 antagonist nutlin-3a was added to the cells and the loss in BRET was observed as PMIγ was displaced from the binding pocket of MDM2. At a 1 μM extracellular peptide concentration, displacement of the peptides from MDM2 was seen only in CMKLR1-expressing cells (Figure 4E). A larger loss of BRET was observed for **3Cy**, again confirming a more efficient cytosolic delivery. Incubation with 10 μM peptides gave similar results, except for an additional unspecific effect in cells without CMKLR1 (Figure 4F). BRET displacement data for the 24 and 48 h time points followed similar trends (Figure S11).

**Figure 4:**
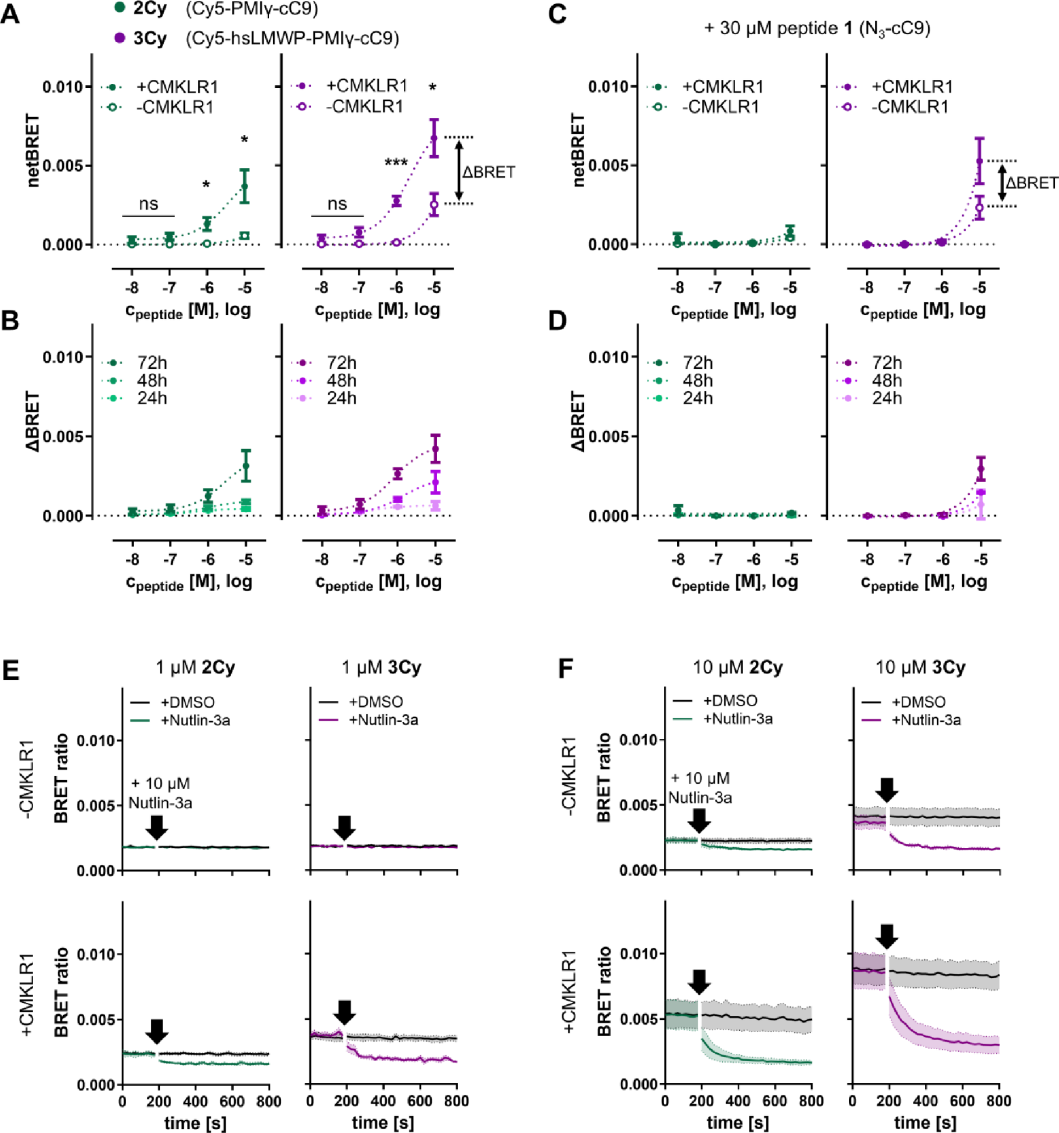
Intracellular bioluminescence resonance energy transfer (BRET) between NanoLuc-MDM2 and Cy5-labeled PMIγ in living cells. (A) Peptides were incubated on HEK293 and HEK293_CMKLR1-eYFP cells for 72 h before BRET was measured. To evaluate the receptor-specific effect, ΔBRET was calculated by subtracting netBRET of cells without CMKLR from netBRET from cells with CMKLR1. Mean ± SEM from three independent experiments performed in duplicate. Statistical comparison of cells with and without CMKLR1 by unpaired *t* test. ***p ≤ 0.001, **p ≤ 0.01, *p ≤ 0.05, ns – not significant. (B) Calculated ΔBRET values after different incubation times of peptides on the cells. (C, D) Same as panels (A) and (B), but peptide uptake was competed by coincubation with 30 μM peptide 1 (N_3_-cC9). (E, F) Displacement of PMIγ peptides from MDM2 after 72 h internalization in a kinetic BRET measurement. After 3 min, 10 μM of the small molecule MDM2 antagonist Nutlin-3a was added and the loss of NanoLuc-MDM2/Cy5-PMIγ BRET was observed. Mean (bold line) ± SEM (dotted lines) from three independent experiments is shown.

### Delivery of Unlabeled Peptide Cargos

Lastly, to demonstrate the biological activity in the absence of a fluorescent label, a further experiment was performed in which BRET between NanoLuc-MDM2 and p53-HaloTag was displaced by the unlabeled peptides **2** and **3**. Here, both peptides demonstrated CMKLR1-selective effects at extracellular concentrations above 1 μM (Figure 5). Again, peptide **3** reached a higher effect compared to that of peptide **2**, but neither achieved full competition of the p53/MDM2 interaction. Considering the 90 nM *K*_d_ of the PMIγ/MDM2 interaction, the amount of BRET displacement correlates well with the intracellular peptide concentrations from the FCS measurement (∼50–200 nM). Thus, these data serve as a very successful proof of concept to show that receptor-mediated shuttling of D-peptide cargos can reach intracellular concentrations high enough to achieve a biological effect, even when receptor activity and target binding affinity are both in the upper nanomolar range. There seems to be significant potential for further optimizations, as PMIγ could be replaced by the much more potent PMIδ peptide (*K*_d_ = 220 pM) that has been reported previously,^49^ and affinity toward CMKLR1 could likely be optimized by improving cargo size and electric charge.

**Figure 5:**
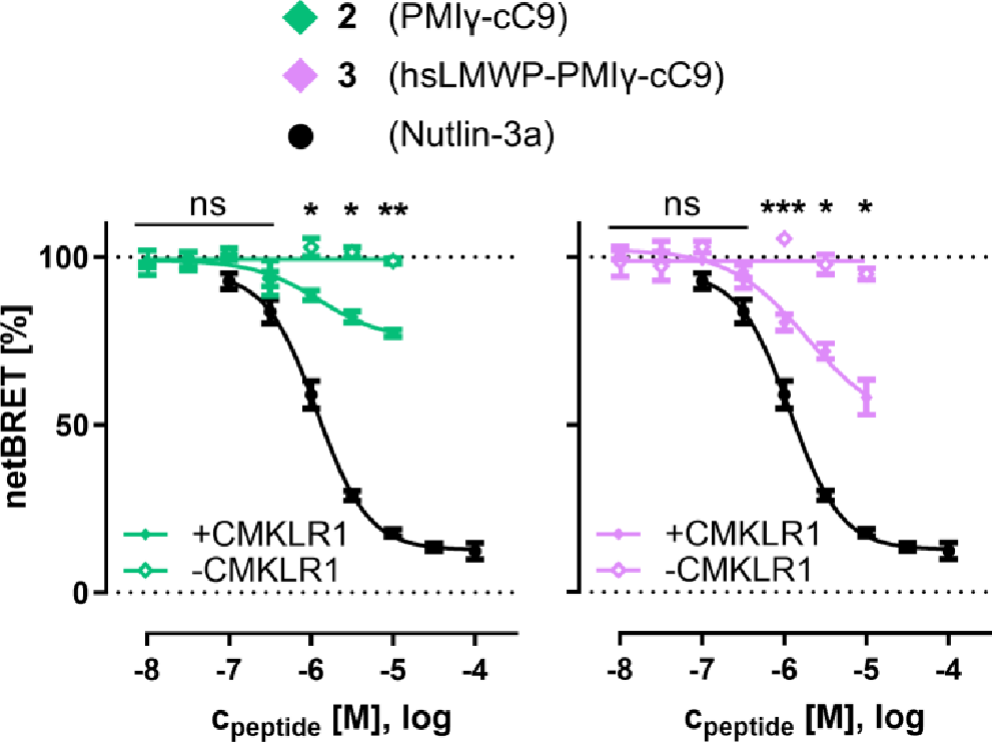
Inhibition of bioluminescence resonance energy transfer (BRET) between NanoLuc-MDM2 and p53-HaloTag by cytosolic peptides. Peptides were incubated on HEK293 and HEK293_CMKLR1-eYFP cells for 72 h, before BRET was measured. Nutlin-3a was incubated on HEK293 cells for 24 h before BRET was measured. Mean ± SEM from two independent experiments performed in duplicate. Statistical comparison of cells with and without CMKLR1 by unpaired *t* test. ***p ≤ 0.001, **p ≤ 0.01, *p ≤ 0.05, ns – not significant.

## Conclusions

In this study, we successfully demonstrated the cytosolic delivery of the D-amino acid peptide PMIγ through a receptor-mediated shuttling system, achieving biologically relevant concentrations exceeding 100 nM. Our data demonstrate that fluorescence correlation spectroscopy can be used as a platform to investigate receptor-mediated shuttle systems and aid in the development of improved endosomal escape modalities. Intracellular binding and biological activity in BRET assays correlated well with the measured FCS data and underscored that the delivered peptides successfully inhibited the p53/MDM2 interaction by binding MDM2. Although efficient and faster targeted delivery remains challenging, the data presented here prove that the achievable concentrations can be sufficient to engage a wide range of potential intracellular targets. These findings have significant implications for the future development of peptide therapeutics addressing intracellular targets and highlight how affinity and stability can be enough to drive a biological effect.

## Supplementary Material



## Notes

Experimental procedures, peptide characterization and additional experimental data (PDF)
