# Supplementary material for: Receptor-Mediated Shuttling of a D‑Amino Acid Peptide Achieves High Nanomolar Cytosolic Concentrations
Source: J Am Chem Soc. 2025 Dec 21;148(1):308–15. doi: 10.1021/jacs.5c12876 (PMC12814185; doi:10.1021/jacs.5c12876)
Supplement: Supplementary file 1 [file ja5c12876_si_001.pdf]

Receptor mediated shuttling of a D-amino acid peptide achieves high nanomolar cytosolic concentrations

Moritz List, Annette G. Beck-Sickinger*

Institute of Biochemistry, Faculty of Life Sciences, Leipzig University, Leipzig, 04103 Germany

Supplementary Information

Experimental section

Peptide synthesis

Alkyne peptides were synthesized in a 15 µmol scale using an orthogonal 9-fluorenylmethoxycarbonyl/tert-butyl (Fmoc/tBu) solid-phase synthesis strategy on a Syro I automated peptide synthesizer (MultiSynTech) using or TGR Rink amide resin (NovaSyn). Coupling reactions were performed twice with 8 eq of Fmoc-protected amino acid (Iris Biotech), 8 eq ethyl cyanohydroxyiminoacetate (oxyma, Iris Biotech) and 8 eq *N,N*-diisopropylcarbodiimide (DIC, Iris Biotech) in *N,N*-dimethylformamide (DMF, VWR) for 30 min. Fmoc-deprotection was achieved with 40% piperidine in DMF (v/v) for 3 min followed by 20% piperidine (Sigma) in DMF (v/v) for 10 min. Ethylene glycol linkers were introduced by reaction with 2 eq 12-(9-Fluorenylmethyloxycarbonylamino)-4,7,10-trioxa-dodecanoic acid (Fmoc-NH-PEG(3)-COOH, Iris Biotech), 1.9 eq hexafluorophosphate azabenzotriazole tetramethyl uronium (HATU, Novabiochem), and 2 eq *N,N*-diisopropylethylamine (DIPEA, Carl Roth). Fluorescent labelling was achieved by reaction with 2 eq Sulfo-Cy5 NHS ester (Lumiprobe), and 1.9 eq DIPEA in DMF for 6h. Peptides were cleaved from the resin with 90% TFA (Sigma), 7% thioanisole (Sigma), and 3% ethanedithiol (Sigma) (v/v/v) for 3 h at RT. Crude peptides were precipitated from diethyl ether at −20 °C for 2 h, washed, and collected by centrifugation. Peptides were purified by RP-HPLC on a 3.6 µm Aeris Peptide XB-C_{18} 100 Å preparative HPLC column (Phenomenex). Purity was analyzed by analytical RP-HPLC on two different columns and peptide identity was confirmed by ESI-Orbitrap MS (Orbitrap Elite, ThermoFisher Scientific). The azide peptide **1** (N_3-cC9) was obtained from Dr. Isabell Kemker (Bayer AG) as a generous gift.

Copper-catalyzed azide-alkyne cycloaddition (CuAAC)

CuAAC was performed in 60% DMSO/water (v/v) with a final concentration of 10 mM of the alkyne peptide. All solutions were degassed with argon before use. 1 eq alkyne peptides and 1.5 eq azide peptide **1** were first dissolved in dimethyl sulfoxide (DMSO, Sigma). 8 eq copper sulfate pentahydrate (Carl Roth) and 16 eq tris(3-hydroxypropyltriazolylmethyl)amine (THPTA, Iris Biotech) in water were heated to 50 °C for 5 min and added to the peptides. 16 eq sodium ascorbate (Sigma) in water were added and the reaction mix was incubated for 90-120 min at 50 °C before addition of 10 eq ethylenediaminetetraacetic acid (EDTA, Sigma) stopped the reaction. The crude reaction mix was then diluted in 20% ACN/water (v/v) and purified by preparative RP-HPLC as described above.

Fluorescence polarization (FP)

20 nM of Cy5-labeled peptides were added to dilutions of human MDM2 (Recombinant Human GST-MDM2/HDM2, R&D Systems) in FP buffer (50 mM tris(hydroxymethyl)aminomethane (Tris, Carl Roth), 150 mM sodium chloride (Sigma), 1 mM EDTA (Sigma), 0.01% (v/v) Tween20 (Sigma), pH 7.4) and incubated for 30 min at room temperature. Fluorescence polarization was then measured in a Tecan Spark plate reader (λ_{ex} = 640 nm, λ_{em} = 670 nm). Measurements were performed twice in technical triplicates. Data was then fit with Prism 10 (GraphPad) using non-linear regression with the following solution to the quadratic binding equation 1 to obtain K_d:

(Equation 1)

$$FP = FP_{min} + (FP_{max} - FP_{min}) * \left(c_{peptide} + c_{MDM2} + K_d - \frac{\sqrt{\left(c_{peptide} + c_{MDM2} + K_d\right)^2 - 4 * c_{peptide} * c_{MDM2}}}{2 * c_{peptide}} \right)$$

Cell culture

HEK293 cells (ACC305, Deutsche Sammlung von Mikroorganismen und Zellkulturen DSMZ) were cultivated in Dulbecco's modified Eagle's medium (DMEM)/Ham's F12 (1:1 (v/v), Biowest) with 15% heat-inactivated fetal bovine serum (FBS, Sigma). Stably transfected HEK293_CMKLR1-eYFP cells (previously described by Fischer et al.[1]) were cultivated in DMEM/Ham's F12 (1:1 (v/v), Biowest) with 15% FBS and 100 µg/mL hygromycin (InvivoGen). Stably transfected COS-7_CMKLR1-eYFP_ $\Delta 6G_{\alpha qi4myr}$ cells (previously described by Lentschat et al.[2]) were cultivated in DMEM with 10% FBS and 200 µg/mL hygromycin. HMEC-1 cells (CRL-3243, American Type Culture Collection ATCC) were cultivated in MCDB131 medium (Gibco) with 10% FBS, 10 ng/mL EGF (Sigma) and 1 µg/mL hydrocortisone (Sigma). MDA-MD231 cells (ACC732, DSMZ) were cultivated in RPMI1640 medium (Biowest) with 10% FBS, 2mM glutamine (Biowest), 10 µg/mL insulin (Sigma). THP-1 cells (ACC16, DSMZ) were cultivated in RPMI1640 with 10% FBS. Cells were maintained at 37 °C, 95% humidity and 5% CO_2. Unless noted otherwise, all incubations with peptides were

performed in DMEM with 10% FBS due to its higher pH buffering capacity compared to DMEM/F12 with 15% FCS.

Calcium flux assay

Calcium flux assay was performed with cells expressing CMKLR1 and the chimeric G protein $\Delta 6G_{\alpha qi4myr}$. The stably transfected COS-7_CMKLR1-eYFP_ $\Delta 6G_{\alpha qi4myr}$ cells were seeded at 25,000 cells per well into poly-D-lysine-coated 96-well plates (black µCLEAR, Greiner). The next day, cells were incubated with 2.3 µM Fluo-2-AM (Abcam) and 0.06% (v/v) Pluronic-F127 (Sigma) in assay buffer (2.5 mM probenecid (Sigma), 20 mM 4-(2-hydroxyethyl)-1-piperazineethanesulfonic acid (HEPES, Sigma) in Hanks' Balanced Salt Solution (HBSS, Biowest), pH 7.5) for 1 h. This solution was replaced by assay buffer, and basal Ca^{2+} signal (λ_{ex} = 485 nm, λ_{em} = 525 nm) was measured for 20 s with a Flexstation 3 plate reader (Molecular Devices). Peptide dilutions were added, and the Ca^{2+} response was measured for 40 s. The maximum signal over baseline was calculated and normalized to peptide **1** (N_3-cC9). Non-linear regression was performed using Prism 10 (GraphPad). Experiments were performed in technical duplicates and at least twice.

Cell viability assay

HEK293 and HEK293_CMKLR1-eYFP cells were seeded at 15,000 cells per well into poly-D-lysine coated 96-well plates (black µCLEAR, Greiner). The next day, dilutions of the Cy5-labeled peptides or Nutlin-3a were added to the cells and incubated for 72 h. Cells were then washed and a resazurin-based cell viability assay was performed. For this, a 1:10 dilution of resazurin solution (Sigma-Aldrich) in DMEM/Ham's F12 (1:1) + 15% FBS without phenol red was added to the cells. After 2h incubation, cell viability was measured using the fluorescence of resorufin (λ_{ex} = 550 nm, λ_{em} = 595 nm). Data was normalized to cells incubated without peptide (100%) and cells treated with ethanol for 3 min before resazurin addition (0%). Experiments were performed in technical triplicates and at least twice.

Fluorescence microscopy

Peptide internalization was evaluated by fluorescence microscopy. HEK293 and stably transfected HEK293_CMKLR1-eYFP cells were seeded at 110,000 cells per well into poly-D-lysine-coated 8-well microscopy slides (ibidi). The next day, 1 µM Cy5-labeled peptides in cell culture medium were added to the cells and incubated for 6 h. Nuclei were counterstained with Hoechst33342 and cells were washed with FluoroBrite™ DMEM (Gibco) with 10% FBS, 25 mM HEPES (pH 7.4) before imaging on an AxioVision Observer.Z1 microscope equipped with an apotome imaging system, an HXP120C light source, and a C-Apochromat 63x/1.20 W objective. All shown microscopy images are representative of at least two independent experiments.

Intracellular peptide stability

2 mL of 1 µM Cy5-labeled peptide in DMEM/Ham's F12 (1:1) + 15% FBS without phenol red were incubated on stably transfected HEK293_CMKLR1-eYFP cells in T25 cell culture flasks for 15 min to 24 h. The cells were washed with cold acidic wash (50 mM glycine, 100 mM sodium chloride, pH 3) and phosphate-buffered saline (PBS, Biowest) and harvested into 300 µl cold PBS with Halt™ Protease inhibitor cocktail (Thermo). Suspended cells were homogenized with zirconia beads on a FastPrep-24 5G system (MP Biomedical) for 30 s and cellular proteins were precipitated from 600 µL ACN/ethanol (1:1) + 0.1% TFA at -20 °C for 2 h. Cell lysates were then filtered through Costar Spin-X tubes, separated by RP-HPLC and isolated peaks were identified by MALDI-ToF MS (Ultraflextreme, Bruker). Experiments were performed at least twice and representative RP-HPLC chromatograms are shown.

Fluorescence correlation spectroscopy (FCS)

Cytosolic delivery was quantified by FCS as has been described previously.[3–5] Stably transfected HEK293_CMKLR1-eYFP cells were seeded at 30,000 cells per well and HMEC-1 cells at 10,000 cells per well into poly-ᴅ-lysine-coated 8-well microscopy slides (ibidi) in their respective cell culture medium. THP-1 cells were resuspended in fresh cell culture medium at 100,000 cells/mL in 500 µL cell culture medium in uncoated 12-well plates. The next day, dilutions of Cy5-labeled peptides were added to the cells and incubated for 72 h. Cell membranes of HMEC-1 and THP-1 cells were stained with 20 µg/mL Alexa Fluor 488-labeled concanavalin A (Invitrogen) for 20 min to enable identification of the cytosol. Nuclei were counterstained with Hoechst33342, and cells were washed with FluoroBrite™ DMEM (Gibco) + 25 mM HEPES (pH 7.4). After washing, THP-1 cells were transferred to poly-ᴅ-lysine-coated 8-well microscopy slides and allowed to sediment and adhere for 1 h. Confocal imaging and FCS were performed at 22 °C on a TCS SP8 FALCON microscope (Leica) with a Plan-Apochromat 40x/1,10 water immersion objective and HyD single molecule detector (Leica). The 638 nm laser line was used for excitation at 0.5% power and fluorescence was detected from 645 to 700 nm. Prior to measurements, the correction collar of the objective was adjusted, and the focal volume was calibrated using a reference solution of 10 nM sulfo-Cy5 (Lumiprobe) in FluoroBrite™ DMEM in an empty well of the microscopy slide. Confocal microscopy was used to identify locations in the cytosol without vesicular compartments for cytosolic FCS measurements. Autocorrelation traces were then collected for 5 s with 10 consecutive repeats at each position. Repeats were averaged and fitted to equation 2 which describes hindered 3D Gaussian diffusion with an additional exponential term included to account for the photoisomerization of sulfo-Cy5 at µs-timescales.[6,7] Here $G(\tau)$ is the autocorrelation at τ, N the average number of molecules in the focal volume, τ_D the average diffusion time through the focal volume, α the anomalous coefficient describing the hindered diffusion, S the shape factor of the focal volume, τ_E the time domain of the additional exponential term and E the fraction of molecules in the exponential term.

(Equation 2)

$$G(\tau) = \frac{1}{N} * \left(1 - E + E * e^{-\frac{\tau}{\tau_E}}\right) * \frac{1}{1 + \left(\frac{\tau}{\tau_D}\right)^{\alpha}} * \sqrt{\frac{1}{1 + S^2 * \left(\frac{\tau}{\tau_D}\right)^{\alpha}}} + G(\infty)$$

For calibration, the effective volume V_{eff} and shape factor S of the focal volume were determined using a reference solution of the free sulfo-Cy5 fluorophore with a known diffusion coefficient. For this, the diffusion coefficient D_{Cy5} at 22 °C was calculated to be 341.6 µm^2/s using equation 3, where D_{Cy5} is 370 µm^2/s at 25 °C,[8] and the dynamic viscosity of water η is 0.954 Pa·s at 22 °C or 0.891 Pa·s at 25 °C respectively.[9]

$$D_{Cy5}(22\,°C) = D_{Cy5}(25\,°C) * \frac{(22\,°C + 273.15) * \eta(25\,°C)}{(25\,°C + 273.15) * \eta(22\,°C)} \quad \text{(Equation 3)}$$

The calibrated shape factor S was generally found between 0.115 and 0.140 and was fixed at 0.125 for all further measurements and calculations. The effective focal volume V_{eff} was calculated using equations 4 and 5 and found to be 1.07±0.10 fL (mean±SD).

$$\omega_0 = 2\sqrt{D_{Cy5} * \tau_D} \quad \text{(Equation 4)}$$

$$V_{eff} = \pi^{\frac{3}{2}} * \omega_0^3 * \frac{1}{S} \quad \text{(Equation 5)}$$

Autocorrelation curves from intracellular measurements were then collected and fitted to equation 2 using a custom MATLAB (MathWorks) script which was based on a script from Garrett Cobb,[10] and reworked to enable compatibility with the files from the Leica microscope.[11] Here, measurements that had inconsistent count rates, low quality fitting ($R^2 < 0.65$), less than 500 counts/s/molecule, diffusion times τ_D under 20 µs or over 20 ms, or an anomalous coefficient α under 0.3 were discarded, the remaining curves were averaged and fitted again weighted by the inverse square of the standard deviation between repeats. A flow chart of the data processing from the MATLAB script is shown in scheme S5. Using these criteria, about 50% of measurements were retained, with slightly fewer successful measurements at lower extracellular peptide concentrations. Accordingly, most discarded data arose from insufficient cytosolic concentrations which did not produce high quality autocorrelation curves. Cytosolic concentrations were then calculated from the successfully fitted curves using equation 6, where N_A is Avogadro's number ($6.022 \cdot 10^{23}$ mol^{-1}). Diffusion coefficients were calculated analogously using equation 7. FCS experiments were performed at least three independent times.

$$c = \frac{N}{N_A * V_{eff}} \quad \text{(Equation 6)}$$

$$D = \frac{V_{eff}^{\frac{2}{3}} * S^{\frac{2}{3}}}{4 * \pi * \tau_D} \quad \text{(Equation 7)}$$

Determination of diffusion coefficients in solution

10 nM dilutions of the Cy5-labeled peptides in Fluorobrite DMEM were measured by FCS to determine their diffusion coefficients in aqueous solution. The diffusion coefficient D of a macromolecule can be estimated

by first calculating its hydrodynamic radius using equation 8, where M is its molecular weight and ρ is the mean density of proteins (1.2 g/cm^3).[12]

$$r = \sqrt[3]{\frac{3 * M}{4 * \pi * N_A * \rho}} \qquad \text{(Equation 8)}$$

From this, the diffusion coefficient can be calculated by the Stokes-Einstein equation (equation 9) where k is the Boltzmann constant (1.381·10^{-23} J/K) and T is the temperature.[13]

$$D = \frac{k * T}{6 * \pi * \eta * r} \qquad \text{(Equation 9)}$$

Determination of relative CMKLR1-surface expression of different cell lines

MDA-MB-231 cells and stably transfected HEK293_CMKLR1-eYFP cells were seeded at 150,000 cells per well and HMEC-1 cells at 50,000 cells per well into poly-ᴅ-lysine coated 96-well plates in their respective cell culture medium. The monocyte-like THP-1 cells were resuspended in fresh cell culture medium at 400,000 cells/mL in 1 mL cell culture medium. A cell-surface ELISA was performed the next day to determine receptor expression. The suspended THP-1 cells were collected by centrifugation at 500 g for 5 min and resuspended in each respective solution for all washing and incubation steps. All cells were first incubated in Opti-MEM (Gibco) for 30 min and the fixed in 2% paraformaldehyde/PBS (w/v) on ice for 1 h. The fixed cells were washed thrice in PBS for 10 min and unspecific binding was blocked by incubation in DMEM/Ham's F12 (1:1) + 15% FBS without phenol red for 1 h at room temperature. Membrane receptors were labeled with 1:100 anti-CMKLR1 antibody (EPR26501-70, abcam) in blocking solution for 1 h. Cells were washed thrice and incubated with 1:1000 secondary mouse anti-rabbit IgG-HRP antibody (sc-2357, Santa Cruz) in blocking buffer for 1 h, and washed again. For detection, cells were incubated with 100 µL 3,3',5,5'-tetramethylbenzidine solution (TMB soluble, EMD Millipore) for 10 min before the reaction was stopped by addition of 100 µL of 0.25 M hydrochloric acid. 100 µL of the solution was transferred to a new plate, the absorption at 450±4.5 nm was measured and signal from cells incubated without primary antibody was subtracted. To account for variations in seeding density and growth rate between cell lines, the number of cells per well was determined with an automated CellDrop cell counter (DeNovix). For THP-1 cells, this was done right before the TMB incubation to account for loss of cells during the repeated centrifugation and resuspension steps. For adherent cell lines, a replicate plate was prepared in parallel to the ELISA and cells were detached with trypsin/EDTA (Biowest) before counting. To obtain relative levels of CMKLR1 per cell (x_{CMKLR1}), absorption data from the ELISA was then divided by the number of cells per well and normalized to the results from the stably transfected HEK293_CMKLR1-eYFP cells. Experiments were performed in technical triplicates and thrice.

Quantification of total cellular peptide uptake of different cell lines

MDA-MB-231 cells and stably transfected HEK293_CMKLR1-eYFP cells were seeded at 165,000 cells per well and HMEC-1 cells at 55,000 cells per well into poly-D-lysine coated 12-well plates in their respective cell culture medium. The monocyte-like THP-1 cells were resuspended in fresh cell culture medium at 100,000 cells/mL in 1 mL cell culture medium. The next day, 1 µM of Cy5-labeled peptides was added to the cells and incubated for 72 h. Cells were then washed with PBS and detached with trypsin/EDTA. THP-1 cells did not require dissociation but were incubated analogously. Suspended cells were washed thrice with Fluorobrite DMEM™ and collected by centrifugation at 500 *g* for 5 min after each step. Finally, cells were resuspended in 300 µM Fluorobrite DMEM™ and cell density was determined with an automated cell counter (Cell Drop, DeNovix). Cell suspensions were then lysed by ultrasonic homogenization (UP200H, Hielscher) for 2 × 10 s on ice to prevent self-quenching of Cy5 due to high local concentrations in vesicles. The amount of Cy5-labeled peptide in the lysate was then determined by fluorescence measurement (λ_{ex} = 630±7.5 nm, λ_{em} = 670±10 nm) using a dilution series of the Cy-labeled peptides in Fluorobrite DMEM™ with known concentrations as a reference. Total peptide uptake per cell (n_{cell}) was then calculated by dividing total peptide in the lysate by the cell density. Experiments were performed thrice.

Calculation of uptake parameters

Uptake relative to receptor expression was calculated according to equation 10, where n_{cell} is the total peptide uptake per cell as quantified by fluorescence measurements, and x_{CMKLR1} is the relative expression of CMKLR1 compared to stably transfected HEK293_CMKLR1-eYFP cells. Values are reported in arbitrary units and normalized to the uptake of peptide **2Cy** in HEK293_CMKLR1-eYFP cells (100%).

$$uptake\ relative\ to\ receptor\ expression = \frac{n_{cell}}{x_{CMKLR1}} \qquad \text{(Equation 10)}$$

Intracellular Cy5-PMIγ/NanoLuc-MDM2 bioluminescence resonance energy transfer (BRET)

HEK293 and stably transfected HEK293_CMKLR1-eYFP cells were seeded at 15,000 cells per well into poly-D-lysine coated 96-well plates (white Nunc MicroWell, Thermo). The next day, cells in each well were transfected with 0.1 µg Transfection Carrier DNA (Promega) and 0.001 µg NanoLuc-MDM2 fusion vector (Promega) using Lipofectamine 3000 (Invitrogen) according to manufactures instructions. Cells were incubated overnight, washed, allowed to recover for 4 h, washed again and dilutions of the Cy5-labeled peptides were added to the cells and incubated for 24,48 or 72 h. For uptake competition experiments, 30 µM of the unlabeled peptide **1** (N3-cC9) were additionally added to each well. After the incubation, excess peptide was washed away thoroughly, and cells were incubated for 4 h before the cell culture medium was replaced by 50 µl Fluorobrite DMEM with Nano-Glo Live Cell Reagent (Promega). After 10 min incubation, luminescence was measured in a Spark multimode plate reader (Tecan), and BRET ratio was calculated by

dividing the Cy5-signal (670±30 nm) by the NanoLuc signal (450±20 nm). netBRET was calculated by subtracting the BRET ratio from cells which were incubated without peptide. To calculate the CMKLR1-specific effect, ΔBRET was calculated by subtracting the netBRET value obtained in cells without CMKLR1 from netBRET obtained in cells with CMKLR1. After the initial readout, a kinetic BRET displacement was performed with the same cells. For this, luminescence was measured every 22 s. After 3 min, 10 µM of the small molecule MDM2 antagonist Nutlin-3a (Sigma) was added to the cells and BRET was measured for another 7 min. Experiments were performed in technical duplicates and three independent times. Non-linear regression and statistical analysis were performed using Prism 10 (GraphPad).

Inhibition of intracellular p53-HaloTag/NanoLuc-MDM2 BRET

Analogous to the intracellular Cy5-PMIγ/NanoLuc-MDM2 BRET experiment, HEK293 and HEK293_CMKLR1-eYFP cells were seeded and transfected with 0.09 µg Transfection Carrier DNA, 0.01 µg p53-HaloTag fusion vector (Promega), and 0.001 µg NanoLuc-MDM2 fusion vector. The transfected cells were then incubated with dilutions of unlabeled PMIγ-peptides for 72 h or with Nutlin-3a for 24 h. During the last 30 min of the incubation, HaloTag TMR Ligand (Promega) was added to the cells to fluorescently label the p53-HaloTag fusion proteins in the cells. Cells were then washed thoroughly, incubated for 4 h in full cell culture medium without peptides, and the cell culture medium was then replaced by 50 µl Fluorobrite DMEM with Nano-Glo Live Cell Reagent. After 10 min incubation, luminescence was measured, and BRET ratio was calculated by dividing the TMR-signal (600±50 nm) by the NanoLuc signal (450±20 nm). netBRET was calculated and normalized to cells without peptide (100%) and cells which were not labeled with HaloTag TMR Ligand (0%). Experiments were performed in technical duplicates and at least twice. Non-linear regression and statistical analysis were performed using Prism 10 (GraphPad).

Figure S1: Copper-catalyzed azide–alkyne cycloaddition (CuAAC) to obtain conjugates of ᴅ-amino acid peptide with cyclic chemerin-9 (cChem9). Reaction scheme and reverse phase high performance liquid chromatography (RP-HPLC) analysis of the reacion mixture shown below.

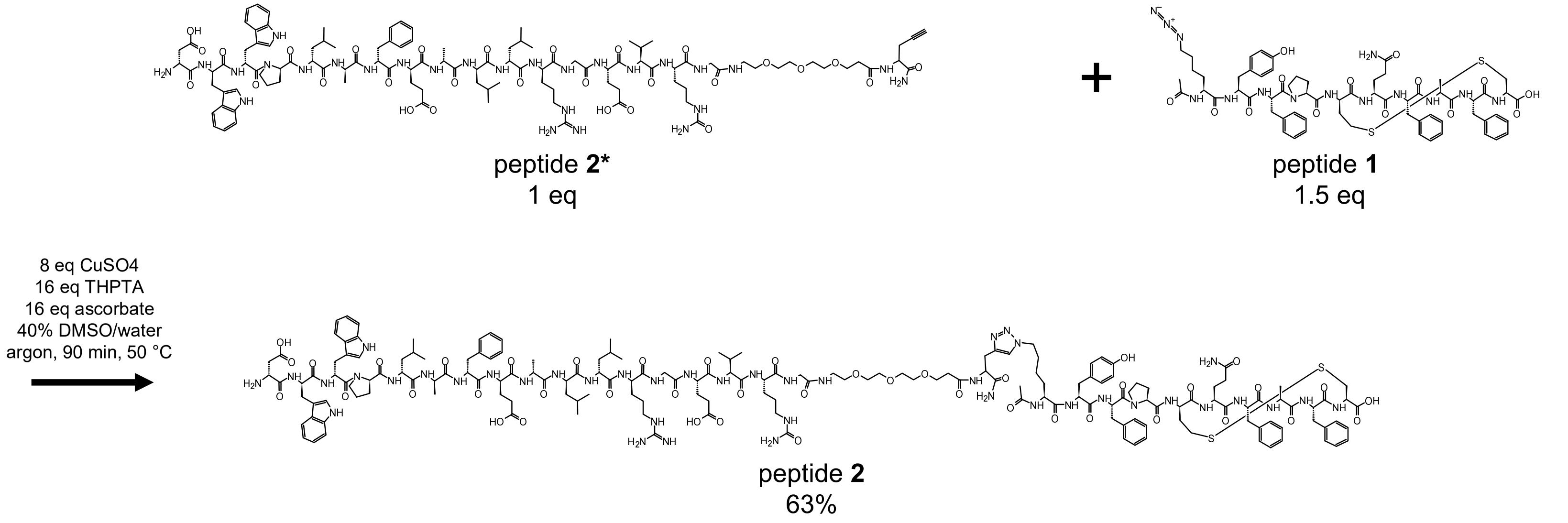

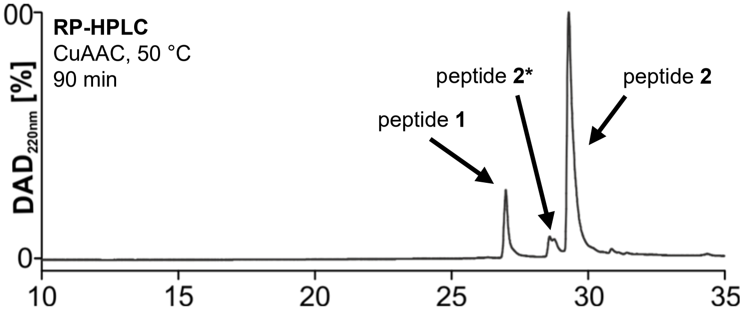

Figure S1: Copper-catalyzed azide–alkyne cycloaddition (CuAAC) to obtain conjugates of D-amino acid peptide with cyclic chemerin-9 (cChem9). Reaction scheme and reverse phase high performance liquid chromatography (RP-HPLC) analysis of the reacion mixture shown below.

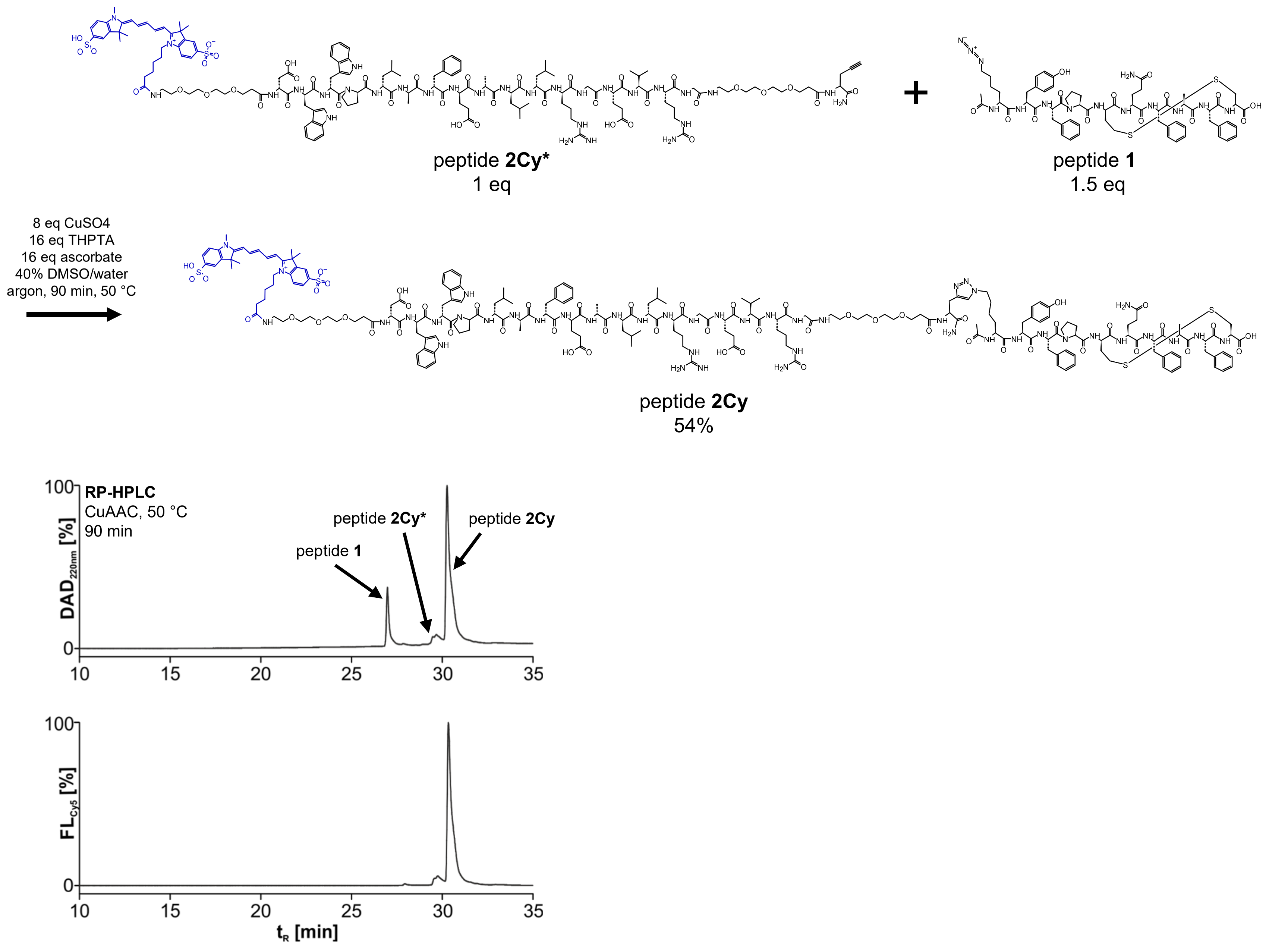

Figure S1: Copper-catalyzed azide–alkyne cycloaddition (CuAAC) to obtain conjugates of D-amino acid peptide with cyclic chemerin-9 (cChem9). Reaction scheme and reverse phase high performance liquid chromatography (RP-HPLC) analysis of the reacion mixture shown below.

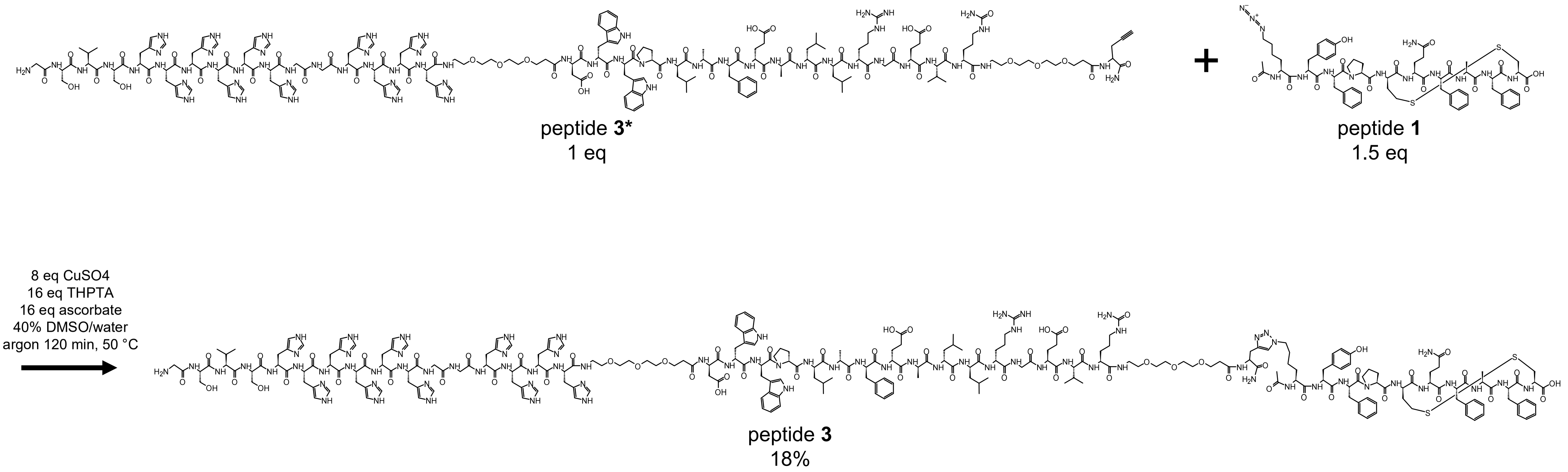

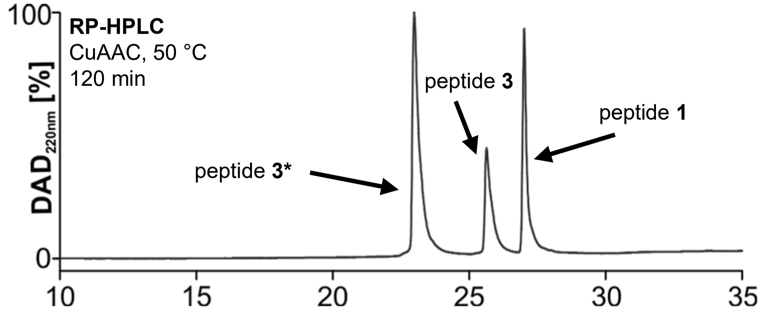

Figure S1: Copper-catalyzed azide–alkyne cycloaddition (CuAAC) to obtain conjugates of D-amino acid peptide with cyclic chemerin-9 (cChem9). Reaction scheme and reverse phase high performance liquid chromatography (RP-HPLC) analysis of the reacion mixture shown below.

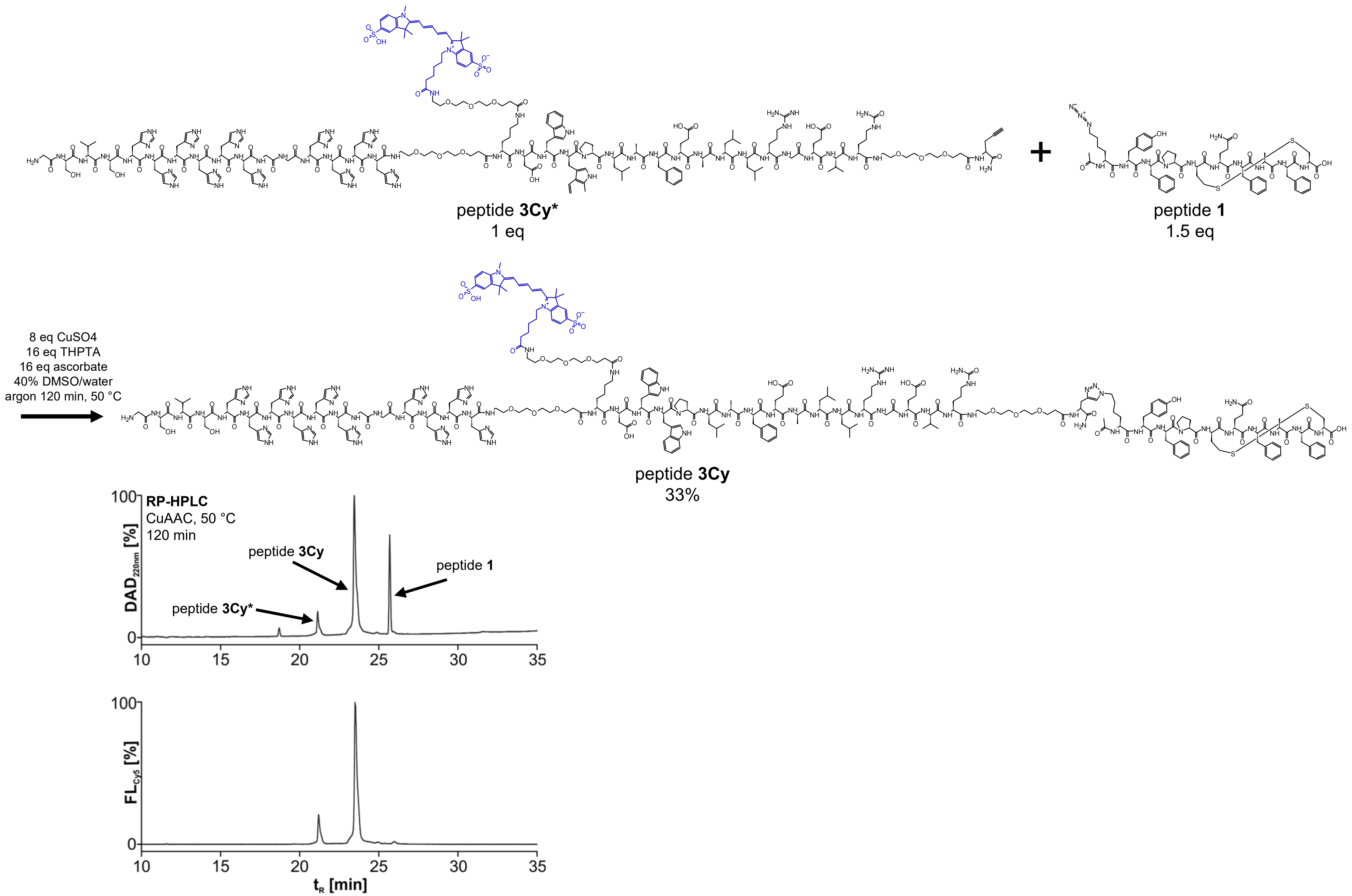

Peptide **1**: N_3-cC9 Ac-K(N_3)YFP-**hcys**-QFAF**C**-OH (M_{mono} = 1332.54 Da)

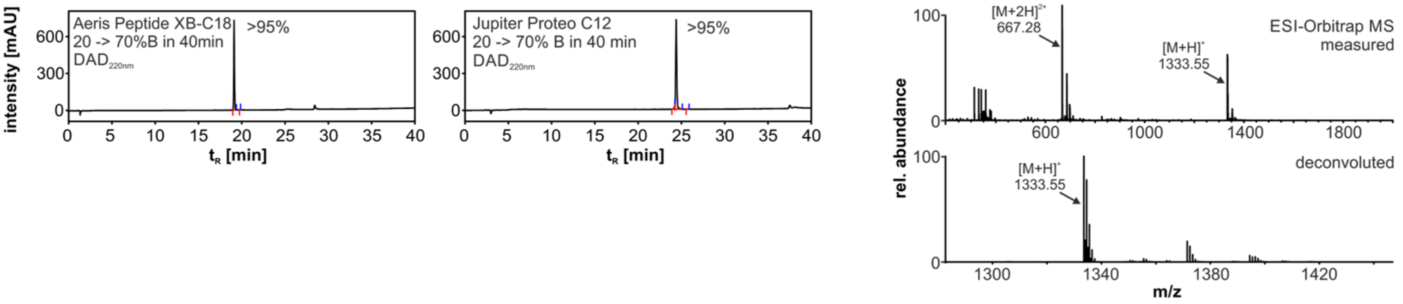

Peptide **2**: PMIγ-cC9 dwwplafeallrGEV-Cit-(EG)$_3$-Pra(cC9)-NH_2 (M_{mono} = 3644.74 Da)

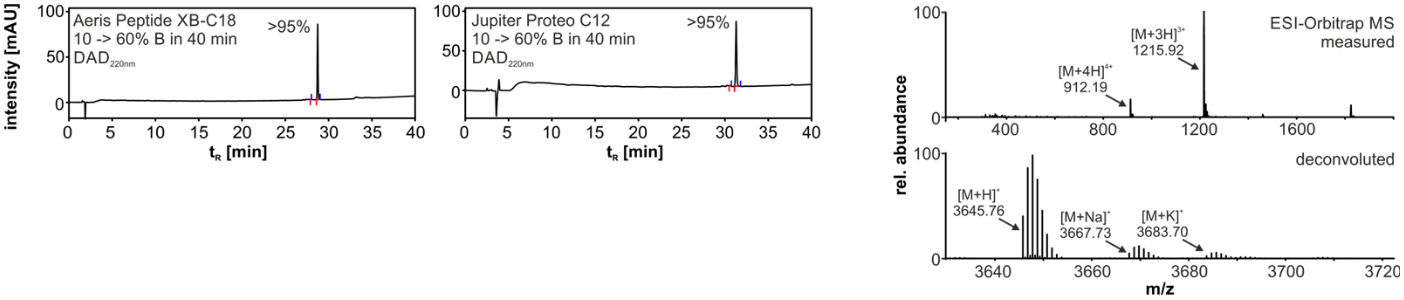

Peptide **2Cy**: Cy5-PMIγ-cC9 Cy5-(EG)$_3$-dwwplafeallrGEV-Cit-(EG)$_3$-Pra(cC9)-NH_2 (M_{mono} = 4472.05 Da)

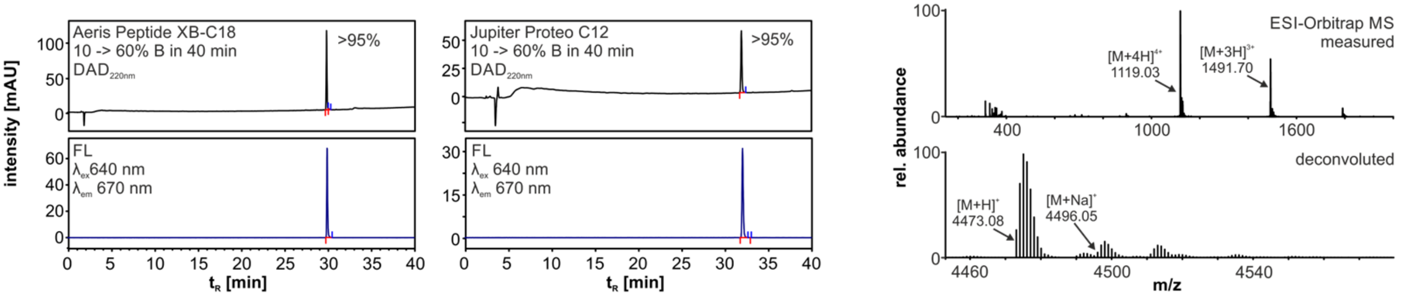

Peptide **3**: hsLMWP-PMIγ-cC9 GSVSHHHHHHGGHHHH-(EG)$_3$-dwwplafeallrGEV-Cit-(EG)$_3$-Pra(cC9)-NH_2 (M_{mono} = 5605.62 Da)

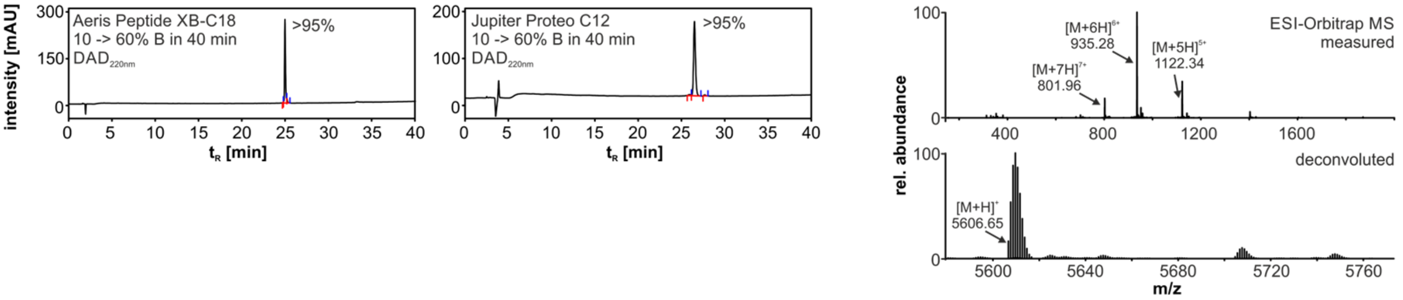

Figure S2. RP-HPLC and ESI-MS Orbitrap analysis of all peptides used in this study. RP-HPLC was performed on Aeris Peptide XB-C18 (Phenomenex, 250 × 4.6 mm, 3.6 µm, 100 Å, flow rate 1.55 ml/min) and Jupiter Proteo C12 (Phenomenex, 250 × 4.6 mm; 4 µm; 90 Å, flow rate 1.00 ml/min) columns using the indicated gradients of solvent A (0.1% trifluoroacetic acid (TFA) in water) and solvent B (0.08% TFA in acetonitrile (ACN)) at 40 °C. For ESI-MS, peptides were dissolved in 0.1% formic acid in 30% ACN/water and measured on a ThermoFisher Orbitrap Elite system. cC9 = cyclic chemerin-9, Cy5 = sulfo-cyanine5, K(N_3) = azidolysine, hcys = D-homocysteine, Cit = citrulline, (EG)$_3$ = triethylenglycol, Pra = propargylglycine, TFA – trifuoroacetic acid

Peptide **3Cy**: Cy5-hsLMWP-PMIγ-cC9 GSVSHHHHHHGGHHHH-(EG)$_3$-k(Cy5-(EG)$_3$)dwwplafeallrGEV-Cit-(EG)$_3$-Pra(cC9)-NH$_2$ (M_{mono} = 6561.02 Da)

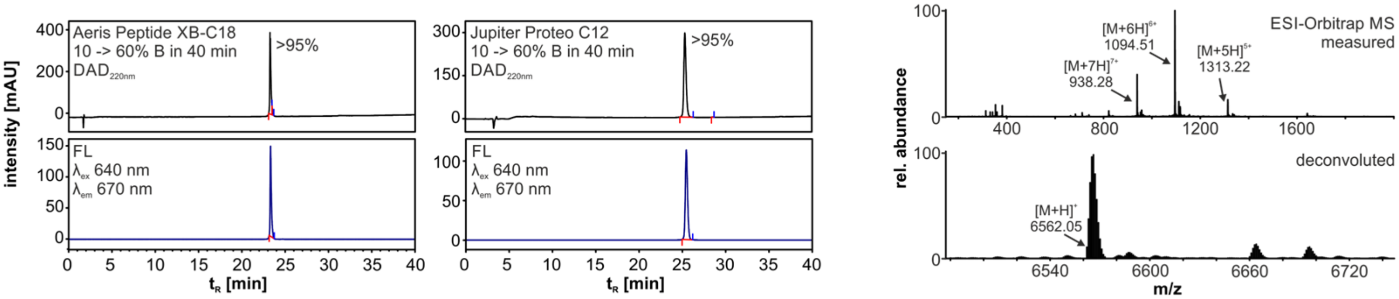

Peptide **4**: r$_9$-PMIγ NH$_2$-rrrrrrrrr-(EG)$_3$-dwwplafeallrGEV-Cit-(EG)$_3$-Pra-NH$_2$ (M_{mono} = 3863.20 Da)

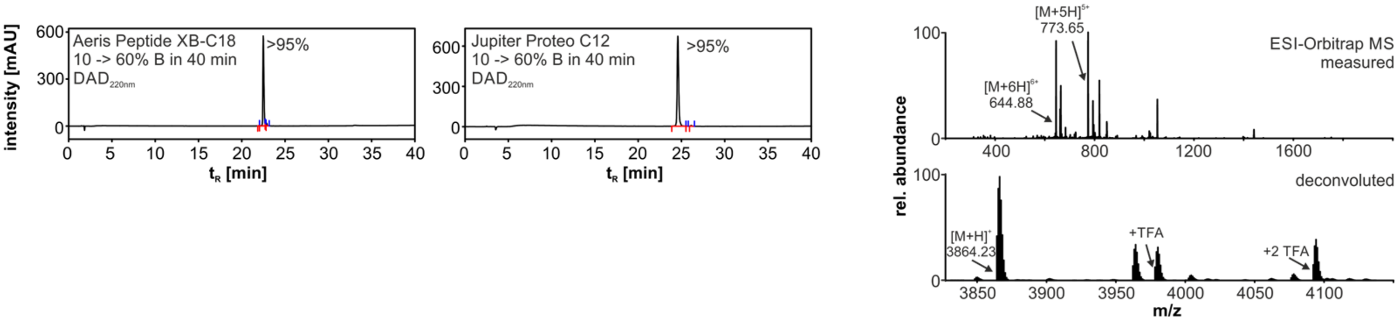

Peptide **4Cy**: Cy5-r$_9$-PMIγ Cy5-(EG)$_3$-rrrrrrrrr-(EG)$_3$-dwwplafeallrGEV-Cit-(EG)$_3$-Pra-NH$_2$ (M_{mono} = 4690.51 Da)

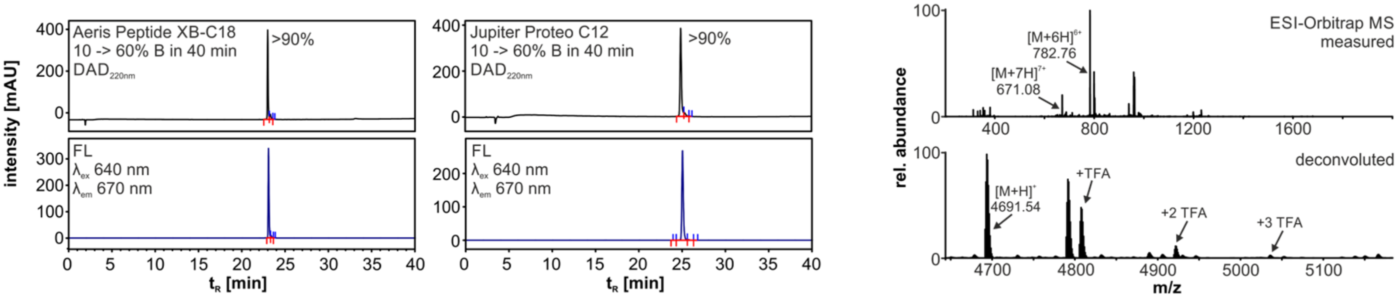

Figure S2. RP-HPLC and ESI-MS Orbitrap analysis of all peptides used in this study. RP-HPLC was performed on Aeris Peptide XB-C18 (Phenomenex, 250 × 4.6 mm, 3.6 µm, 100 Å, flow rate 1.55 ml/min) and Jupiter Proteo C12 (Phenomenex, 250 × 4.6 mm; 4 µm; 90 Å, flow rate 1.00 ml/min) columns using the indicated gradients of solvent A (0.1% trifluoroacetic acid (TFA) in water) and solvent B (0.08% TFA in acetonitrile (ACN)) at 40 °C. For ESI-MS, peptides were dissolved in 0.1% formic acid in 30% ACN/water and measured on a ThermoFisher Orbitrap Elite system. cC9 = cyclic chemerin-9, Cy5 = sulfo-cyanine5, K(N_3) = azidolysine, hcys = ᴅ-homocysteine, Cit = citrulline, $(EG)_3$ = triethylenglycol, Pra = propargylglycine, TFA – trifuoroacetic acid

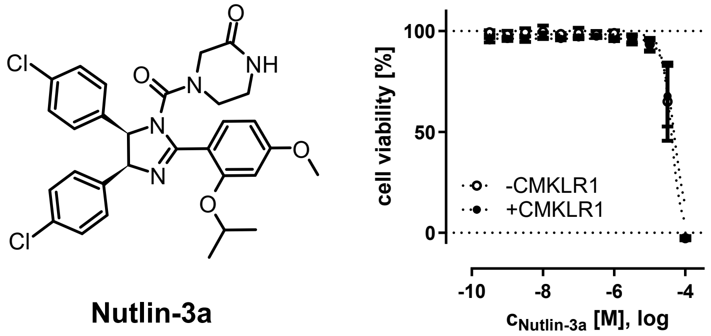

Figure S3. Resazurin based cell viability assay of HEK293 and HEK293_CMKLR1-eYFP cells after 72 h incubation with the p53/MDM2 inhibitor Nutlin-3a. Mean ± SEM from two independent experiments performed in triplicates. p53-unspecific toxicity at 100 µM was previously demonstrated by Liu et al.[24].

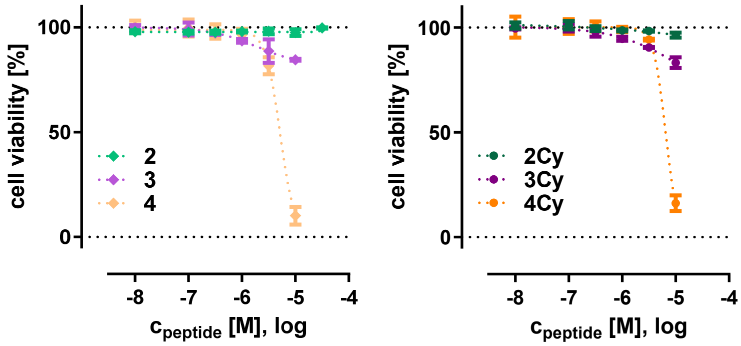

Figure S4. Resazurin based cell viability assay of HEK293_CMKLR1-eYFP cells after 72 h incubation with the PMIγ peptides discussed in this work. Mean ± SEM from two independent experiments performed in triplicates.

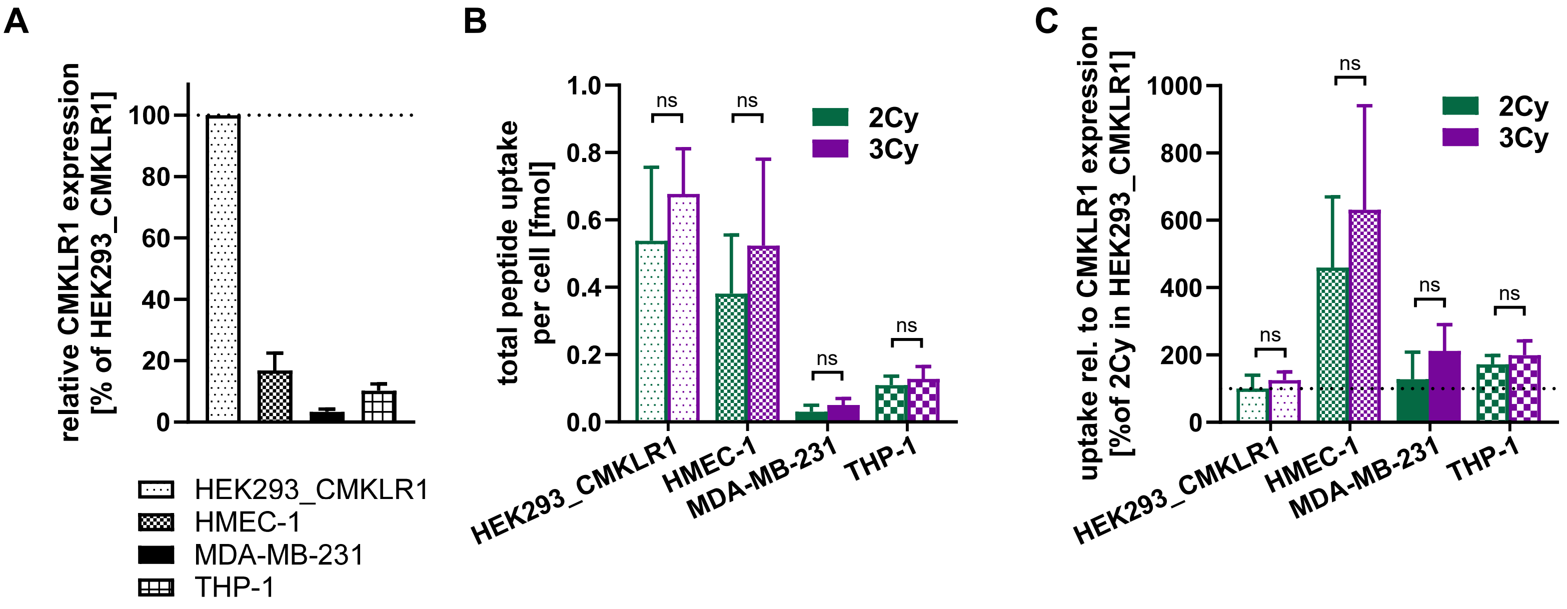

Figure S5. Quantification of receptor-mediated uptake in CMKLR1-expressing cell lines. (A) Cell-surface enzyme-linked immunosorbent assay (ELISA) for the quantification of CMKLR1 expression of different cell lines. ELISA data was divided by the cell number and normalized to the stably transfected HEK293_CMKLR1-eYFP cells. Mean ± SD from three independent experiments performed in triplicates. (B) Total amount of Cy5-labeled peptide taken up by different cell lines after incubation with 1 µM peptide for 72 h. For quantification, cells were detached, counted, lysed by ultrasonic homogenization, and fluorescence was measured in the lysate. Total peptide uptake per cell was calculated from a dilution series of Cy5-labeled peptide with known concentration. Mean ± SD from three independent experiments. (C) Data from figure S5B divided by the relative CMKLR1 expression from figure S5A and normalized to the uptake of **2Cy** in HEK293_CMKLR1-eYFP cells. Statistical analysis by unpaired t test. ***p ≤ 0.001, **p ≤ 0.01, *p ≤ 0.05, ns – not significant

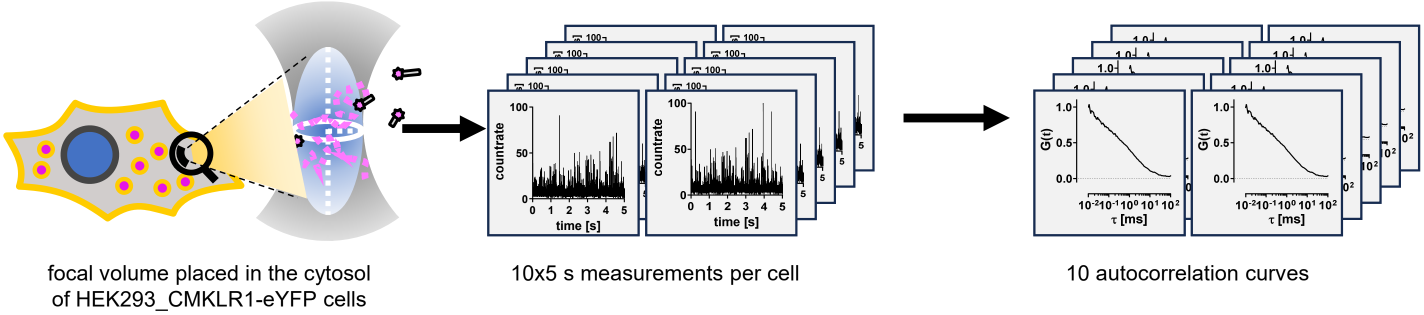

Hindered 3D Gaussian diffusion with additional exponential term

$$G(\tau) = \frac{1}{N} * \left(1 - E + E * e^{-\frac{\tau}{\tau_E}}\right) * \frac{1}{1 + \left(\frac{\tau}{\tau_D}\right)^{\alpha}} * \sqrt{\frac{1}{1 + S^2 * \left(\frac{\tau}{\tau_D}\right)^{\alpha}}} + G_\infty$$

$G(\tau)$	autocorrelation at t	τ_D	diffusion time
N	number of molecules in focal volume	α	anomalous coefficient
E	exponential fraction	S	shape factor
τ_E	exponential fraction time		

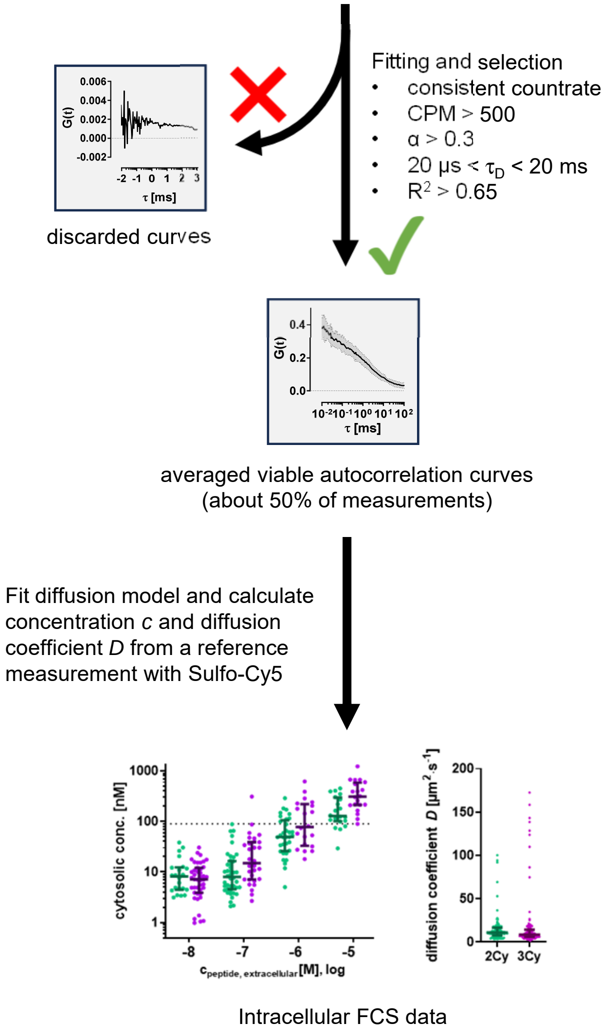

Scheme S6. Data processing and evaluation of fluorescence correlation (FCS) measurements.

Table S7. Descriptive statistics of fluorescence correlation spectroscopy (FCS) data from Figure 3. Measurements were obtained in stably transfected HEK293_CMKLR1-eYFP cells as well as endogenously CMKLR1-expressing HMEC-1 and THP-1 cells. SD – standard deviation, GSD – geometric SD factor, IQR – interquartile range

cell line	N°	name	applied concentration [nM]	mean ± SD [nM]	geometric mean *÷ GSD [nM]	median (IQR) [nM]
HEK293_CMKLR1-eYFP	**2Cy**	Cy5-PMIγ-cC9	10	8.4 ± 7.8	6.0 *÷ 2.2	6.2 (3.1–9.1)
			100	11.6 ± 14.6	7.1 *÷ 2.6	6.8 (3.3–13.0)
			1000	78.6 ± 83.2	44.6 *÷ 2.8	43.1 (19.0–99.4)
			10000	161 ± 128	112 *÷ 2.5	106 (60.2–269)
	**3Cy**	Cy5-hsLMWP-PMIγ-cC9	10	6.6 ± 5.0	4.9 *÷ 2.3	5.5 (3.2–9.5)
			100	23.5 ± 40.2	11.9 *÷ 3.0	10.2 (5.2–32.1)
			1000	138 ± 122	87.7 *÷ 3.0	98.8 (37.8–199)
			10000	308 ± 257	239 *÷ 2.0	228 (157–435)
HMEC-1	**2Cy**	Cy5-PMIγ-cC9	1000	7.8 ± 5.4	6.3 *÷ 1.9	6.4 (3.7–9.3)
	**3Cy**	Cy5-hsLMWP-PMIγ-cC9	1000	20.1 ± 57.2	9.2 *÷ 2.5	7.0 (5.2–12.4)
THP-1	**2Cy**	Cy5-PMIγ-cC9	1000	22.7 ± 25.1	14.5 *÷ 2.6	14.0 (7.1–26.6)
	**3Cy**	Cy5-hsLMWP-PMIγ-cC9	1000	40.3 ± 77.12	23.0 *÷ 2.6	22.6 (12.7–38.5)

Table S8. Predicted and measured diffusion coefficients *D* in solution. Measurements were performed with 10 nM of fluorescent compounds in Fluorobrite DMEM at 22 °C. Data from three independent measurements. Mean and standard deviation are shown.

	name	molecular weigth [Da]	predicted *D* [µm²/s]	measured *D* [µm²/s]
	sulfo-Cy5	778.0	-	341.6 (reference)
**2Cy**	Cy5-PMIγ-cC9	6565.3	198.9	154.2 ± 4.3
	Intracellular cleavage product	2401.8	244.7	-
**3Cy**	Cy5-hsLMWP-PMIγ-cC9	4475.1	175.0	138.5 ± 5.1
	Intracellular cleavage product	2733.2	234.4	-

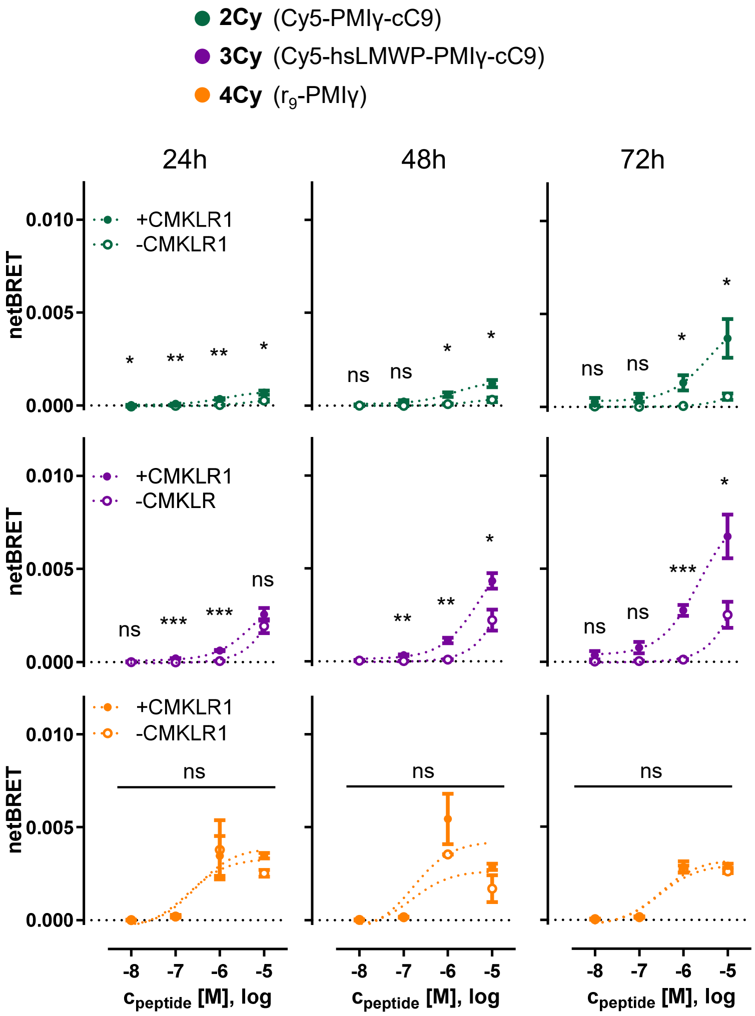

Figure S9. Full dataset of Figure 4 A and B. Intracellular bioluminescence resonance energy transfer (BRET) between NanoLuc-MDM2 and Cy5-labeled PMIγ in living cells. Peptides were incubated on HEK293 and HEK293_CMKLR1-eYFP cells for 24, 48 or 72 h, before BRET was measured. Mean ± SEM from three independent experiments performed in duplicates. Statistical comparison of cells with and without CMKLR1 by unpaired t test. ***p ≤ 0.001, **p ≤ 0.01, *p ≤ 0.05, ns – not significant

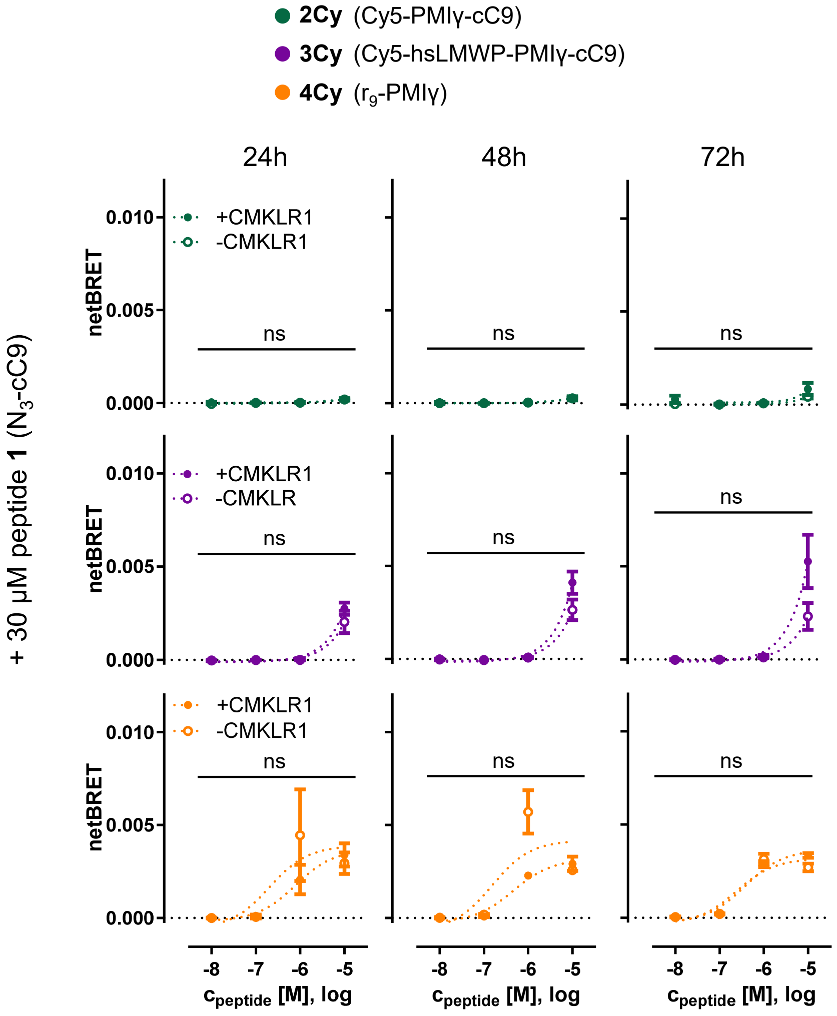

Figure S10. Full dataset of Figure 4 C and D. Intracellular bioluminescence resonance energy transfer (BRET) between NanoLuc-MDM2 and Cy5-labeled PMIγ in living cells. Cy5-labeled peptides were co-incubated with 30 µM peptide **1** (N_3-cC9) on HEK293 and HEK293_CMKLR1-eYFP cells for 24, 48 or 72 h, before BRET was measured. Mean ± SEM from at least two independent experiments performed in duplicates. Statistical comparison of cells with and without CMKLR1 by unpaired t test. ***p ≤ 0.001, **p ≤ 0.01, *p ≤ 0.05, ns – not significant

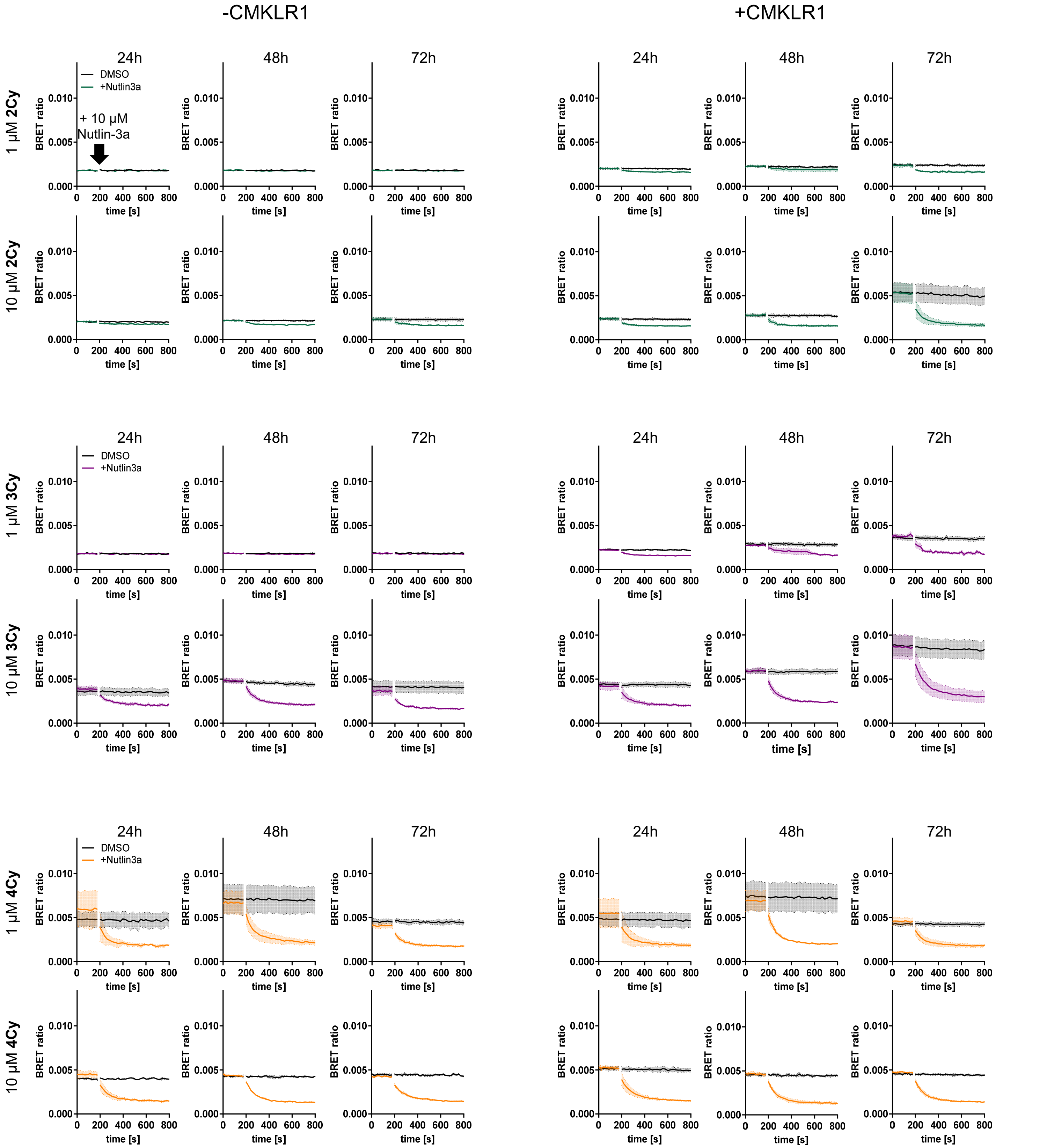

Figure S11. Full dataset of Figure 4 E and F. Intracellular displacement of PMIγ peptides from MDM2 after 24, 48 or 72 h internalization in a kinetic BRET measurement. After 3 min, 10 µM Nutlin-3a was added and the loss of NanoLuc-MDM2/Cy5-PMIγ BRET was observed. Mean (bold line) ± SEM (dotted lines) from at least two independent experiments.

References

(1) Fischer, T. F.; Czerniak, A. S.; Weiß, T.; Zellmann, T.; Zielke, L.; Els-Heindl, S.; Beck-Sickinger, A. G. Cyclic Derivatives of the Chemerin C-Terminus as Metabolically Stable Agonists at the Chemokine-like Receptor 1 for Cancer Treatment. *Cancers* **2021**, *13* (15), 3788.

(2) Lentschat, H.; Liessmann, F.; Tydings, C.; Schermeng, T.; Stichel, J.; Urban, N.; Schaefer, M.; Meiler, J.; Beck-Sickinger, A. G. Hederagenin is a Highly Selective Antagonist of the Neuropeptide FF Receptor 1 that Reveals Mechanisms for Subtype Selectivity. *Angewandte Chemie International Edition* **2025**, *64* (6), e202417786.

(3) Knox, S. L.; Steinauer, A.; Alpha-Cobb, G.; Trexler, A.; Rhoades, E.; Schepartz, A. Quantification of protein delivery in live cells using fluorescence correlation spectroscopy. *Methods in Enzymology* **2020**, *641*, 477–505.

(4) Steinauer, A.; LaRochelle, J. R.; Knox, S. L.; Wissner, R. F.; Berry, S.; Schepartz, A. HOPS-dependent endosomal fusion required for efficient cytosolic delivery of therapeutic peptides and small proteins. *Proceedings of the National Academy of Sciences of the United States of America* **2019**, *116* (2), 512–521.

(5) Wissner, R. F.; Steinauer, A.; Knox, S. L.; Thompson, A. D.; Schepartz, A. Fluorescence Correlation Spectroscopy Reveals Efficient Cytosolic Delivery of Protein Cargo by Cell-Permeant Miniature Proteins. *ACS Central Science* **2018**, *4* (10), 1379–1393.

(6) Huang, Z.; Ji, D.; Xia, A.; Koberling, F.; Patting, M.; Erdmann, R. Direct observation of delayed fluorescence from a remarkable back-isomerization in Cy5. *Journal of the American Chemical Society* **2005**, *127* (22), 8064–8066.

(7) Widengren, J.; Schwille, P. Characterization of Photoinduced Isomerization and Back-Isomerization of the Cyanine Dye Cy5 by Fluorescence Correlation Spectroscopy. *The Journal of Physical Chemistry A* **2000**, *104* (27), 6416–6428.

(8) Loman, A.; Dertinger, T.; Koberling, F.; Enderlein, J. Comparison of optical saturation effects in conventional and dual-focus fluorescence correlation spectroscopy. *Chemical Physics Letters* **2008**, *459* (1-6), 18–21.

(9) Huber, M. L.; Perkins, R. A.; Laesecke, A.; Friend, D. G.; Sengers, J. V.; Assael, M. J.; Metaxa, I. N.; Vogel, E.; Mareš, R.; Miyagawa, K. New International Formulation for the Viscosity of H2O. *Journal of Physical and Chemical Reference Data* **2009**, *38* (2), 101–125.

(10) LaRochelle, J. R.; Cobb, G. B.; Steinauer, A.; Rhoades, E.; Schepartz, A. Fluorescence correlation spectroscopy reveals highly efficient cytosolic delivery of certain penta-arg proteins and stapled peptides. *Journal of the American Chemical Society* **2015**, *137* (7), 2536–2541.

(11) Stephen23. *Natural-Order Filename Sort*. https://www.mathworks.com/matlabcentral/fileexchange/47434-natural-order-filename-sort (accessed 2025-07-18).

(12) Siegel, A. P.; Baird, M. A.; Davidson, M. W.; Day, R. N. Strengths and weaknesses of recently engineered red fluorescent proteins evaluated in live cells using fluorescence correlation spectroscopy. *International Journal of Molecular Sciences* **2013**, *14* (10), 20340–20358.

(13) Einstein, A. Über die von der molekularkinetischen Theorie der Wärme geforderte Bewegung von in ruhenden Flüssigkeiten suspendierten Teilchen. *Annalen der Physik* **1905**, *322* (8), 549–560.
